# Rebooting Synthetic Phage-Inducible Chromosomal Islands: One Method to Forge Them All

**DOI:** 10.34133/2020/5783064

**Published:** 2020-05-11

**Authors:** Rodrigo Ibarra-Chávez, Andreas F. Haag, Pedro Dorado-Morales, Iñigo Lasa, José R. Penadés

**Affiliations:** ^1^Institute of Infection, Immunity and Inflammation, College of Medical, Veterinary and Life Sciences, University of Glasgow, Glasgow, UK; ^2^Laboratory of Microbial Pathogenesis, Navarrabiomed, Complejo Hospitalario de Navarra-Universidad Pública de Navarra (UPNA), Instituto de Investigación Sanitaria de Navarra (IDISNA), 31008 Pamplona, Spain

## Abstract

Phage-inducible chromosomal islands (PICIs) are a widespread family of mobile genetic elements, which have an important role in bacterial pathogenesis. These elements mobilize among bacterial species at extremely high frequencies, representing an attractive tool for the delivery of synthetic genes. However, tools for their genetic manipulation are limited and timing consuming. Here, we have adapted a synthetic biology approach for rapidly editing of PICIs in *Saccharomyces cerevisiae* based on their ability to excise and integrate into the bacterial chromosome of their cognate host species. As proof of concept, we engineered several PICIs from *Staphylococcus aureus* and *Escherichia coli* and validated this methodology for the study of the biology of these elements by generating multiple and simultaneous mutations in different PICI genes. For biotechnological purposes, we also synthetically constructed PICIs as Trojan horses to deliver different CRISPR-Cas9 systems designed to either cure plasmids or eliminate cells carrying the targeted genes. Our results demonstrate that the strategy developed here can be employed universally to study PICIs and enable new approaches for diagnosis and treatment of bacterial diseases.

## 1. Introduction

In recent years, we have extensively studied the biology and genetics of a novel class of very widespread, chromosomally located, mobile elements, the phage-inducible chromosomal islands (PICIs) [1–3]. PICIs are phage satellites, intimately related to certain temperate (helper) phages, whose life cycles they parasitize. Following infection by a phage or SOS induction of a prophage, PICI genomes excise from the bacterial chromosome, using PICI-encoded integrases (*int*) and excision functions (*xis*) [3–5], they replicate extensively using their own replicons [3, 6] and are efficiently packaged into infectious particles composed of phage virion proteins [3, 7–9]. This preferential packaging occurs at the expense of helper phage packaging, which is blocked by the PICIs using several elegant and sophisticated strategies of molecular piracy [2, 10–12].

In addition to their fascinating life cycle, PICIs have raised curiosity in the scientific community because of their importance as key players driving bacterial evolution and virulence. Of note is the fact that PICIs are clinically important because they carry and disseminate virulence and antibiotic-resistance genes [13]. For example, the prototypical and best-characterized members of the PICI family, the *Staphylococcus aureus* pathogenicity islands (SaPIs), carry and spread toxin-encoding genes between bacteria, including one that can cause toxic shock syndrome in humans [14]. Interestingly, it has been recently proposed that these elements could be used as an alternative to phages and antibiotics to combat *S. aureus* infections [15].

While the interest in characterizing PICIs is clear, their study has been partially limited by the lack of efficient tools to easily manipulate these elements in different species. Currently, manipulation of the PICI genomes requires multiple steps including cloning of the desired constructs in *Escherichia coli* using shuttle vectors, which will be introduced into the specific strains carrying the PICIs. Then, the genes of interest will be introduced or deleted from the PICIs by double crossover [3, 12, 16–18].

In order to solve this problem, we explored the possibility that some of the methods currently used to manipulate phages could be easily adapted to engineer PICIs [19]. Recombinant *E. coli* phage genomes have been successfully assembled in yeast using a yeast artificial chromosome [20]. This process is called rebooting, where reactivation of a synthetic phage genome takes place in the appropriate host cell after being assembled in yeast or *in vitro*. To do that, the authors amplified the entire phage genome of interest in different PCR fragments that partially overlap (>30 bp overlapping). The first and last fragments of the phage genome are amplified with primers that carry “arms” that have homology with a yeast artificial chromosome (YAC) fragment, which may be obtained by PCR or any other suitable method. The different fragments are (including the viral genome and the YAC) transformed into yeast, and gap repair joins each fragment to the adjacent one templated by the homology regions at the end of each fragment, yielding a full phage genome cloned into a replicative yeast plasmid. The generated YAC-phage genome is purified from the yeast and transformed into *E. coli*, where the synthetic phage will replicate. Then, the cells are lysed with chloroform, and supernatants containing the infective phage particles are mixed with overnight cultures of natural-host bacteria for each phage, to promote phage replication and plaque formation. However, PICIs cannot be reactivated in the same way as the phages because they do not produce functional infective particles in the absence of their helper phage. This difference in their life cycle makes some of the strategies used to engineer phages, including the use of bacterial L-forms [21], not applicable to reboot PICIs. Another limitation is that PICI-encoded integrases are quite promiscuous facilitating integration of these elements in aberrant places in the absence of their cognate *att*B sites [22]. Thus, a circular YAC-PICI element containing the structure of a packaged PICI would probably promote the integration of this element into the yeast genome. An additional obstacle that should be tackled relates to the fact that while the yeast-based approach allows rapid phage genome engineering of lytic *E. coli* phages, its use with other classes of mobile genetic elements, or with phages from Gram-positive bacteria, has not yet been demonstrated.

**Figure 1:**
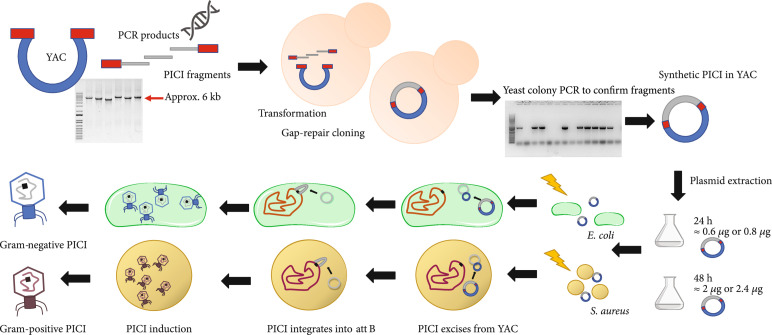
PICI assembly and rebooting workflow. For the assembly of a YAC-PICI, a PCR fragment containing the yeast replicon and selection marker from pAUR112 is required. Additionally, PCR products containing the PICI regions to assemble in the YAC vector by gap-repair cloning are used. All PCR fragments present, at their ends, partially overlapping regions. The final product will mirror the PICI structure in the chromosome. YAC-PICI DNA is extracted from the yeast and directly transformed into competent cells of the host bacterium. Then, the PICI excises from the YAC and integrates into its corresponding attachment site located on the chromosome. The whole process can be followed through the selective marker present in the island. For rebooting the island, the induction of its cognate helper phage is needed.

We hypothesized, however, that it should be possible to assemble synthetic PICI genomes into the YAC, followed by a subsequent transfer and reactivation of the YAC-PICI DNA directly into those species of interest. To do this, and to solve the aforementioned problems, we designed a strategy in which the PICIs will be assembled in the YAC mirroring the structure they have when they are integrated into the bacterial chromosome, rather than the genomic structure they have when they are packaged in infective particles. We hypothesized that this strategy would avoid aberrant YAC-PICI integration in the yeast while, after transformation of the YAC-PICI DNA into the species of interest, this generated genomic structure will allow excision of the element from the YAC and the subsequent integration of the PICI into the bacterial chromosome (Figure 1). To facilitate this, the YAC contains not just the PICI genome but also the chromosomal DNA flanking the PICI in the bacterial genome (see scheme in Figure S1). If true, this strategy will significantly impact the field by providing a new method to easily manipulate PICIs from different species, including those from relevant Gram-positive and Gram-negative pathogens.

## 2. Materials and Methods

### 2.1. Bacterial Strains and Growth Conditions

All strains used in this study are listed in Table S1. Single colonies from the desired strain were grown overnight in the appropriate media supplemented with antibiotics, where required. Bacterial cells were stored as 15% (v/v) glycerol (Fisher Scientific) stocks at -80°C from overnight cultures. *S. aureus* strains were routinely grown on tryptic soy agar (TSA) or in tryptic soy broth (TSB) (Oxoid) in an orbital shaker at 80 or 120 rpm at 30°C or 37° C, respectively. Antibiotics and chromogenic substrates used included erythromycin (10 *μ*g ml^-1^, Sigma-Aldrich), tetracycline (3 *μ*g ml^-1^, Sigma-Aldrich), and X-gal (80 *μ*g ml^-1^, Roche). *E. coli* strains were routinely grown on Luria-Bertani (LB, Sigma-Aldrich) agar (LBA) plates or in LB liquid medium grown in an orbital shaker at 80 or 120 rpm at 30°C or 37 ° C, respectively. Antibiotics (Sigma-Aldrich) and chromogenic substrates used to maintain transformed plasmids and grow selectively were ampicillin (100 *μ*g ml^-1^ Sigma-Aldrich), chloramphenicol (20 *μ*g ml^-1^ Sigma-Aldrich), and tetracycline (20 *μ*g ml^-1^ Sigma-Aldrich).

### 2.2. Plasmid Construction

KAPA High Fidelity DNA polymerase (Sigma-Aldrich) was used to obtain PCR products for use in cloning. PCR products were purified directly from the PCR reaction mix using the QIAquick® PCR Purification Kit (Qiagen), following manufacturer’s instructions. DNA concentrations were established using a Nanodrop 1000 spectrophotometer (Thermo Fisher Scientific).

The plasmids used in this study (Table S2) were constructed by cloning PCR products, amplified with the oligonucleotides listed in Table S3 (Sigma-Aldrich), into the appropriate vectors [23]. The cloned plasmids were verified by Sanger sequencing (Eurofins Genomics). Plasmids used for PICI rebooting were transformed into the host cells by following the protocol described by Monk et al. [24]. The *P_cad_-cas9-rsaE* and *P_cad_-cas9-∅* sequences were obtained from pFREE [25] and pCN51 [26]; *cas9-mecA* and *cas9-∅* sequences were obtained from pRIC10 and pRIC13; *P_bla_-gfpmut2* sequence was obtained from pCN68 [26]; *P_bla_-bgaB* sequence was obtained from pMAD [17]; *P_tet_-cas9-ndm1* and *P_tet_-cas9-∅* were obtained from pRC319-*∅* and pRC319 [27].

### 2.3. Yeast Assembly

Cells of *S. cerevisiae* strain BY23849 were inoculated into 20 ml of 2X YPAD media (Sigma-Aldrich) and incubate at 30°C, 210 RPM overnight. On a 250 ml flask with 50 ml of 2X YPAD, 5 ml of overnight culture were added and incubated at 30 °C, 210 RPM until the cell density has reached $2.0\times 10^{7}$ cells ml^-1^ or OD_600_ 1.0. This should make up to 12 transformations but can be scaled up. For each transformation, 3-4 ml of cells were harvested by centrifugation at max speed (>6,000 × g) for 30 sec. Cells were resuspended and washed twice in 0.1 M lithium acetate (Sigma-Aldrich) (LiAC) and centrifuged at max speed for 30 sec.

For each pellet, 260 *μ*l of PEG 50% (Fisher Scientific) w/v, 36 *μ*l of 1.0 M LiAC (Sigma-Aldrich), and 50 *μ*l of salmon sperm single-stranded DNA (2 mg ml^-1^ Sigma Aldrich) were added. Carrier DNA needs to be at room temperature for 5 min prior to use. Following this, 14 *μ*l of a mixture of DNA and MilliQ gradient H_2_O containing YAC and PCR fragments (~250 ng of YAC PCR and ~500 ng of each PCR fragment) were added to the transformation mixture. Note that the volume can exceed the 14 *μ*l mentioned above if the concentration of fragments is lower than 500 ng *μ*l^-1^. Cells can also be frozen and stored using storing solution (5% glycerol Fisher Scientific, 10% DMSO Sigma-Aldrich) and incubating them in a slow freezing container for 4 h at -80°C.

Cells were vortexed vigorously to resuspend the pellet and then incubated at 42°C for 45 min. Cells need to be mixed periodically. Cells were then centrifuged at >6,000 × g for 3 min, and the supernatant was removed. Finally, 200 *μ*l of MilliQ grade water was added carefully to resuspend the pellet and cells subsequently plated onto appropriate synthetic dropout media (SD) plates (see supplementary material and methods for more details). Plates were incubated at 30°C for 3 days.

### 2.4. Yeast-Colony PCR and Plasmid Extraction

Between 10 and 15 colonies were restreaked onto SD media plates and incubated at 30°C overnight. Yeast colony PCRs were performed using primers from one region to the other in order to generate a product that proves the gap repair between two PCR fragments. From each isolated colony, a smear was added with a 10 *μ*l tip onto an Eppendorf microfuge tube and microwaved for 5 min at full power placing the tubes at the edge of the microwave plate. After this, tubes were left on ice for 5 min and the PCR reaction mix was added to each sample.

Once positive colonies were identified, these were inoculated into 20 ml of SD media in a 100 ml flask and incubated for 48 h at 30°C with shaking at 210 rpm. To harvest the plasmids, cells were spun down at >6,000 × g for 5 min, resuspended into 500 *μ*l of lyticase buffer (1 M sorbitol Fisher Scientific and 0.1 M Na_2_EDTA pH 7.5 Thermo-Fisher), and split into two tubes. Then 20 *μ*l l of lyticase solution (Lyticase from Arthrobacter luteus 2.5 *μ*g *μ*l^-1^ Sigma-Aldrich, 1.2 M sorbitol Thermo-Fisher and 10 mM sodium phosphate pH 7.5 Fisher Scientific) was added, and samples were incubated for 2 h at 37°C and 120 rpm. After incubation, samples were centrifuged at >6,000 × g for 3 min, and plasmids were harvested using Qiagen miniprep extraction kit following the manufacturer’s protocol.

### 2.5. PICI Rebooting

For PICI rebooting, competent cells were transformed with plasmids (Table S2) and after electroporation incubated for 2 h at 37°C and 120 rpm. Generally, >1 *μ*g of plasmid DNA is used for a successful transformation in *E. coli* and >2 *μ*g for *S. aureus* electrocompetent cells [16, 24].

### 2.6. Induction and Titration

For *S. aureus*, an overnight culture in TSB (Sigma-Aldrich) was diluted 1 : 50 in TSB and cultured in a shaking incubator at 37°C and 120 rpm until 0.2-0.3 OD_540_. PICIs and phages were then induced by adding mitomycin C (1 mg ml^-1^) at a final concentration of 2 *μ*g ml^-1^. For *E. coli* PICIs and phages, an overnight culture in LB media (Sigma-Aldrich) was diluted in 1 : 50 and cultured in a shaking incubator at 37°C and 150 rpm until 0.15-0.17 OD_600_. PICIs and phages were then induced by adding mitomycin C (1 mg ml^-1^ Sigma-Aldrich) at a final concentration of 1 *μ*g ml^-1^. The cultures were incubated at 30°C and 80 rpm for 3-4 hours. Generally, cell lysis occurred 4-5 h postinduction. To store lysates, the solution was filtered through a 0.2 *μ*m filter (Minisart® single use syringe filter unit, hydrophilic and nonpyrogenic, Sartorious Stedim Biotech), and the phage stock was stored at 4°C or -80°C.

The PICI derivatives used in this work contained a *tet*M, *erm*C, or *cat* antibiotic resistance cassette. These markers allow for the selection of the PICI, on media supplemented with the appropriate antibiotic. Transduction titering assays were performed in *S. aureus* strain RN4220 as recipient for all SaPIs and in *E. coli* using strain 594 as a recipient for the EcCICFT073 islands. A 1 : 50 dilution of an overnight culture was prepared and grown until OD_540_ or $\text{O}{\text{D}}_{600}=1.4$ was reached. Strains were infected using 1 ml of the recipient culture with the addition of 100 *μ*l of phage lysate serial dilutions, prepared in phage buffer, and cultures were supplemented with CaCl_2_ to a final concentration of 5 mM before incubation for 30 min at 37°C. This incubation allows the PICI to infect the acceptor strain. After incubation, 3 ml of top agar (media +3% agar) at 55°C was added and immediately poured over the surface of a plate containing selective antibiotic and necessary nutrients. For *S. aureus*, TSA plates with antibiotics were used for the selective culture of the successfully transduced bacteria with SaPIs. For *E. coli*, LB plates with antibiotics were used for the selective culture of the successfully transduced bacteria with Gram-negative PICIs. After the top agar had solidified (15-20 min), the plates were flipped and incubated at 37°C for 24 h. The number of colonies formed was counted, and the colony-forming units (CFU per ml) were calculated.

An overnight culture of the appropriate recipient strain was diluted 1 : 50 with fresh medium and grown until 0.3-0.4 OD_540_ or OD_600_. The phage lysate was set-up as serial dilutions using phage buffer (1 mM NaCl Fisher Scientific, 0.05 M Tris pH 7.8 Fisher Scientific, 1 mM MgSO_4_, 4 mM CaCl_2_ Fisher Scientific). In a sterile test tube, 100 *μ*l of the recipient cells was added with 100 *μ*l of serial dilutions of phage lysates in phage buffer and incubated at room temperature for 10 min. Then, 3 ml of Phage Top Agar (PTA) (20 g l^-1^ nutrient broth n°2, Oxoid; 3.5 g l^-1^ agar; 10 mM CaCl_2_ Fisher Scientific) at 55°C was added to the tube and immediately poured over the surface of a Phage Base Plate (PB) (20 g l^-1^ nutrient broth n°2, Oxoid; 7 g l^-1^ agar; 10 mM CaCl_2_ Fisher Scientific). When the top agar was solidified after 15-20 min, the plates were flipped and incubated at 37°C overnight. The PBA plates were left at room temperature to dry before being incubated at 37°C for 24 h. The number of plaques formed was counted, and the plaque-forming units (PFU per ml) were calculated.

### 2.7. Capsid Precipitation

Following phage-PICI induction, 1 ml was taken from each filtered lysate to then treat it with DNase (1 *μ*g ml^-1^ Sigma-Aldrich) and RNase (1 *μ*g ml^-1^ Sigma-Aldrich) at room temperature for 30 min. NaCl was added to a final concentration of 0.5 M, and samples were incubated on ice for 1 h. After incubation, samples were transferred into 2 ml microfuge tubes containing 0.25 g of PEG 8000 (Fisher Scientific) and incubated at 4°C overnight. Samples were then centrifuged at 11,000 × g for 10 min at 4°C. The supernatant was discarded and tubes were left to dry for 5 min. Pellets were then resuspended with 100 *μ*l of phage buffer for 1 h. To lyse the capsids, 100 *μ*l of lysis buffer (90 *μ*l H_2_O, 9.5 *μ*l SDS 20% CaCl_2_ Fisher Scientific, 4.5 *μ*l proteinase K 20 mg ml^-1^ Sigma-Aldrich) was added, and samples were incubated at 55°C for 30 min. DNA extraction was then performed by phenol-chloroform extraction and ethanol precipitation. Finally, pellets were resuspended in 75 *μ*l of MilliQ grade H_2_O to then be processed for southern blot.

### 2.8. Southern Blot

Phage-capsid bulk DNA and SaPI-capsid DNA were separated by agarose gel electrophoresis by running samples on 0.7% agarose gel at 30 V, overnight. The DNA was transferred to Nylon membranes (Hybond-N 0.45 mm pore size filters; Amersham Life Science) using standard methods. DNA was detected using a DIG-labeled probe (Digoxigenin-11-dUTP alkali-labile; Roche) and anti-DIG antibody (Anti-Digoxigenin-AP Fab fragments; Roche), before washing and visualization. The primers used to obtain the DIG-labeled probes are listed in Table S3.

### 2.9. SaPI Interference

An overnight culture of RN4220 recipient strains containing the SaPIbov1 variants (strains JP1996, JP20777 to JP20783) were diluted 1 : 50 with fresh medium and grown until 0.3-0.4 OD_540_ or OD_600_. In a sterile test tube, 250 *μ*l of the recipient cells was added with 8 ml of Phage Top Agar (PTA) (20 g l^-1^ nutrient broth n°2, Oxoid; 3.5 g l^-1^ agar; 10 mM CaCl_2_ Fisher Scientific) and immediately poured over the surface of a Phage Base Plate (PB) (20 g l^-1^ nutrient broth n°2, Oxoid; 7 g l^-1^ agar; 10 mM CaCl_2_ Fisher Scientific). When the top agar was solidified after 15-20 min, the plates were flipped and 10 *μ*l of serial dilutions of phage lysate obtained from strains RN10616 and RN451 was spotted onto the solidified lawn. The PBA plates were left at room temperature to dry before being incubated at 37°C for 24 h. The number of plaques formed was counted, and the plaque-forming units (PFU) were calculated.

### 2.10. Killing and Plasmid Curing Assays

A 1 : 50 dilution of an overnight culture was prepared and grown until 1.0 OD_600_ was reached (approx. $4\times 10^{8}$ CFU ml^-1^ for *S aureus* and $8\times 10^{8}$ CFU ml^-1^ for *E. coli*). Cultures were then diluted to obtain a cell density of ~10^5^ CFU ml^-1^. For the rebooted SaPIbov2 versions with CRISPR-Cas9 (JP20279, JP20280, JP21058, and JP21059), titers were normalized to ~10^6^ TFU ml^-1^ to use an MOI of 10. For killing assays, infected *S. aureus* cells were plated on TSA with tetracycline to observe directly the killing effect of strains containing the target. For plasmid curing, cells were plated on TSA with tetracycline to measure the proportion of cells transduced with the synthetic SaPIbov2 and on TSA containing both tetracycline and erythromycin to measure the proportion of cells cured of pCN51 with and without the target (JP13894 and JP17110). For the rebooted EcCFT073 island containing CRISPR-Cas9 (strains JP20690 and JP20691), titers were normalized to ~10^6^ TFU ml^-1^ to use a MOI of 10. *E. coli* cells were plated either on LB with chloramphenicol to measure the proportion of cells transduced with the synthetic PICI or on LB containing both chloramphenicol and tetracycline to measure the proportion of cells cured of pRIC with and without the target (JP17120 and JP17462).

To examine the curing of the plasmids by Cas9 deployment and activity, the ratio of resistance conferred by plasmids to total transductants was calculated using the number of TFUs of each sample over the number of TFUs from cells carrying the control plasmid and transduced with PICIs containing a Cas9-∅ array.

### 2.11. Quantification and Statistical Analysis

All statistical analyses were performed as indicated in the figure legends using the GraphPad Prism 6.01 software, where “n” represents the number of independent experiments.

## 3. Results

### 3.1. Assembly and Rebooting of Synthetic SaPIs

Since SaPIs are the prototypical and best-characterized members of the PICI family, they were used as our first model to test the assembly of synthetic variants in yeast. As a proof of concept, we tried to assemble and reboot the parent SaPIbov1 *tst*::*tet*M, which has been extensively used to study the biology of these elements [18, 28–31]. Note that this SaPI carries a tetracycline resistance cassette (*tet*M) inserted into the toxic shock syndrome gene (*tst*), which facilitates our studies.

Primers with homology overhangs of ≥30 bp were designed to amplify SaPIbov1 (Figures 1 and S1). Since this SaPI is ~15 kb in size, 3 different PCR fragments were designed to amplify the entire element. Each region was chosen according to their function in the SaPI cycle [1]. Region 1 contained the SaPI regulatory and replication modules, region 2 contained the interference and packaging modules, while region 3 contained the accessory (virulence) module. Thus, the use of these regions will allow us to generate in the future chimeric and new SaPI variants. The 5${}^{\prime }$ region of the first fragment and the 3${}^{\prime }$ region of the last fragment carried homology overhangs with the YAC (pAUR112) fragment containing the essential genes for selection and assembly in yeast. Using the method described here, all three SaPIbov1 *tst*::*tet*M and YAC fragments were transformed into *S. cerevisiae* BJ5464 cells to assemble the synthetic SaPI genome. Colonies of *S. cerevisiae* were restreaked and analyzed by colony PCR to confirm the presence of SaPIbov1. For the PCR experiments, we used primers that amplify and detect the recombination region of each two adjoining fragments. Moreover, the presence of the first and last fragments in the YAC was confirmed using external primers to the YAC recombination sites together with fragment-specific primers. Next, the YAC::SaPIbov1 *tst*::*tet*M DNA was purified, electroporated into competent *S. aureus* RN4220 cells, and the transformed cells grown onto TSA plates containing tetracycline. After transformation with the YAC::SaPIbov1 *tst*::*tet*M DNA, and in support of our initial idea, tetracycline-resistant *S. aureus* colonies were detected, suggesting SaPIbov1 *tst*::*tet*M was able to excise from the YAC and integrate into the bacterial chromosome. The presence of the entire SaPIbov1 *tst*::*tet*M element was confirmed by colony PCR employing primers that bind internally and externally to the SaPI *att*C sites to verify integration (See Table S3).

**Figure 2:**
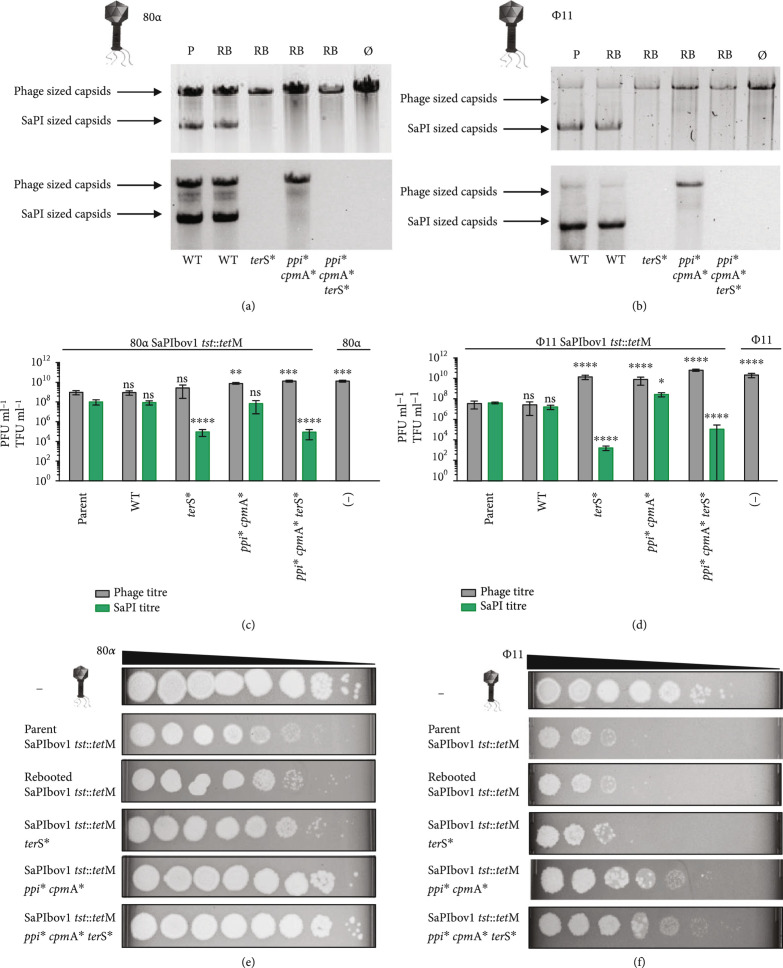
Effects of phage packaging and SaPI transfer on SaPIbov1 engineered mutations. Upper panel: The 80*α* (a) or *ϕ*11 (b) mediated induction, replication, and packaging of the islands was analyzed by analysis of the DNA extracted from the purified SaPI and phage lysates. In these experiments, two bands are observed, corresponding to the phage-sized or SaPI-sized capsids. The lower panel is a Southern blot using a probe for the SaPIbov1 *tet*M cassette. Sample P represents the parent strain containing the helper phage and SaPIbov1 *tst*::*tet*M while samples RB represent the rebooted versions of SaPIbov1 WT, SaPIbov1 *ter*S${}^{*}$, SaPIbov1 *ppi*${}^{*}$*cpm*A${}^{*}$, and SaPIbov1 *ppi*${}^{*}$*cpm*A${}^{*}$*ter*S${}^{*}$. Null (∅) represents a lysate generated from a strain containing only the helper phage as a control. (c, d) The lysates of each SaPIbov1 version obtained after induction of the helper phages 80*α* (c) or *ϕ*11 (d) were analyzed for phage titer (PFU ml^-1^) and SaPIbov1 transduction titer (TFU ml^-1^), using RN4220 as recipient strain. The SaPIbov1-mediated phage interference was tested by infecting strains containing the different SaPIbov1 derivative islands with lysates of phage (e) 80*α* or (f) *ϕ*11. Statistical analysis was performed using two-way ANOVA followed by Dunnett’s multiple comparisons test with the parent strain 80*α* SaPIbov1 *tst*::*tet*M or *ϕ*11 SaPIbov1 *tst*::*tet*M as controls and as reference for all comparisons, respectively (n=6±SD). Adjusted p values for phage titers using helper phage 80*α*, WT ns p=0.9998, *ter*S${}^{*}$ ns p=0.5386, *ppi*${}^{*}$*cpm*A${}^{*}$${}^{**}p=0.0071$, *ppi*${}^{*}$*cpm*A${}^{*}$*ter*S${}^{*}$${}^{***}p=0.0007$ and (-) ${}^{***}p=0.0008$; and for SaPI titers WT ns p=0.9997, *ter*S${}^{*}$${}^{****}p<0.0001$, *ppi*${}^{*}$*cpm*A${}^{*}$ ns p=0.7993, *ppi*${}^{*}$*cpm*A${}^{*}$*ter*S${}^{*}$${}^{****}p<0.0001$. Adjusted p values for phage titers using helper phage *ϕ*11, WT ns p=0.9611, *ter*S${}^{*}$${}^{****}p<0.0001$, *ppi*${}^{*}$*cpm*A${}^{*}$${}^{****}p<0.0001$, *ppi*${}^{*}$*cpm*A${}^{*}$*ter*S${}^{*}$${}^{****}p<0.0001$ and (-) ${}^{****}p<0.0001$; and for SaPI titers WT ns p=0.4029, *ter*S${}^{*}$${}^{****}p<0.0001$, *ppi*${}^{*}$*cpm*A${}^{*}$${}^{*}p=0.0283$, *ppi*${}^{*}$*cpm*A${}^{*}$*ter*S${}^{*}$${}^{****}p<0.0001$.

To confirm the functionality of the synthetic element, a strain carrying the engineered SaPIbov1 *tst*::*tet*M was lysogenized with phage 80*α*, which activates the SaPIbov1 excision-replication-packaging cycle [30]. The strain was induced with mitomycin C (MC), and the phage and SaPI titers were determined and compared to those obtained with the lysogenic strain for phage 80*α* harboring the original SaPIbov1 *tst*::*tet*M. Both islands were indistinguishable in their ability: (i) to replicate after phage 80*α* induction (Figure 2(a)), (ii) to be packaged and transferred to a new recipient strain (Figures 2(b) and 2(c)), and (iii) to block phage reproduction (Figures 2(d) and 2(e)), validating our strategy and confirming that no deleterious mutations arose during the assembly or rebooting processes.

We next tested the ability of this method to generate single, double, or even triple simultaneous mutations in SaPIbov1 *tst*::*tet*M. Specifically, we generated a SaPIbov1 *tst*::*tet*M carrying a mutation in *ter*S (JP20779), one carrying a double mutation in *ppi* and *cpm*A (JP20781), and a final one carrying a triple mutation in *ter*S, *ppi*, and *cpm*A (JP20783). The *ter*S, *ppi*, and *cpm*A genes were selected because they have defined roles in the SaPI life cycle: the SaPI-encoded TerS protein is required for specific SaPI packaging [32], Ppi binds to the phage-encoded TerS protein blocking phage packaging [11], while CpmA expression is required for the production of the small SaPI sized capsids [32, 33]. Figure S2 shows the strategy used to generate the different synthetic PICIs carrying the mutations of interest. Overlapping PCR fragments were produced to introduce two amber stop codons followed by a restriction site (for screening). Using classic approaches, the generation of a triple SaPIbov1 *tst*::*tet*M mutant would be highly time-consuming (several months), requiring several rounds of cloning and mutagenesis. In contrast, if functional, this synthetic biology strategy will allow the production of several mutations in one week.

All SaPIbov1 *tst*::*tet*M mutants were readily assembled in yeast (strains JP20109, JP20733, JP20735, and JP20737) and mobilized into *S. aureus*. Next, the different SaPIbov1-positive cells were lysogenized with phages 80*α* (strains JP21212-JP21215) and *ϕ*11 (strains JP21216-JP21219), and the SaPI cycle analyzed after induction of the prophages with MC. Thus, while all the islands replicate as the original SaPIbov1 *tst*::*tet*M (Figure 2(a)), the transfer of the elements carrying a mutation in the SaPI *ter*S gene was significantly reduced relative to that observed for the original island, and to the same level as that observed for the previously characterized SaPIbov1 *tst*::*tet*M *ter*S mutant [32] (Figures 2(c) and 2(d)). Moreover, the islands carrying the mutation in the *cpm*A gene were unable to generate SaPI-sized (small) capsids (Figures 2(a) and 2(b)), while the SaPIs carrying the double *ppi/cpm*A mutation were unable to interfere with the reproduction of phages 80*α* and *ϕ*11 (Figures 2(e) and 2(f)). For phage *ϕ*11, smaller plaques were observed compared to the reproduction of phage *ϕ*11 on a lawn of RN4220, suggesting that the other mechanisms of interference still have an effect on the reproduction of this phage [33]. When inducing the double (*ppi/cpm*A) or the triple (*ppi/cpm*A/*ter*S) SaPIbov1 mutants, the phage titers were identical to that observed after induction of helper phages 80*α* and *ϕ*11 from a SaPIbov1-negative strain. Taken together, our results confirm that SaPI assembly in yeast is an extraordinary and easy method to gain insights into the biology of these elements.

### 3.2. Assembly and Rebooting of Engineered SaPIs

**Figure 3:**
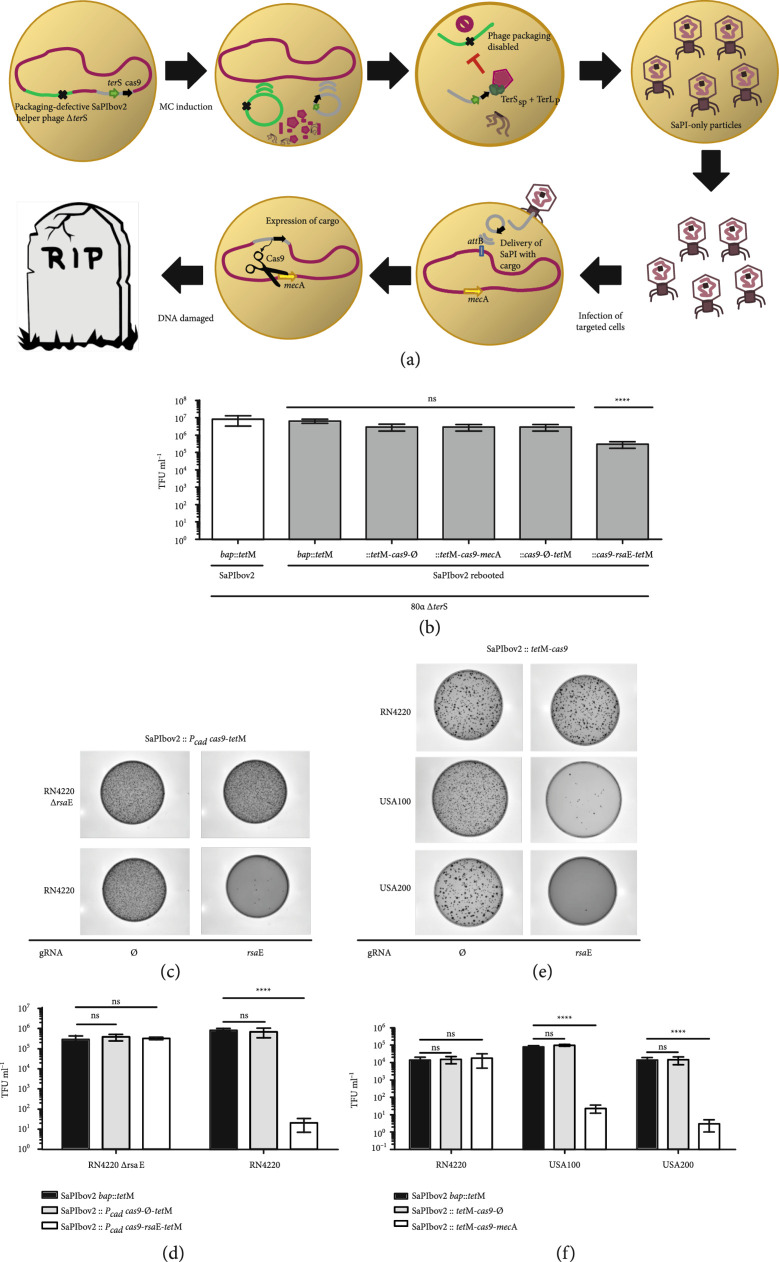
Engineered SaPIs with CRISPR-Cas9 for targeted therapy. (a) The synthetic SaPI exploits the packaging machinery of its temperate phage to pack and deliver a CRISPR-Cas9 (black arrow) cargo into other bacteria. After induction with MC and activation of the ERP cycle, both phage (light green) and SaPI (grey) excise from the bacterial (red) chromosome to be packed into viral particles. Only the SaPI-DNA is packed with its cognate TerS (green arrow) and the TerL of the phage, while the phage-DNA packaging is disabled due to the deletion of the phage TerS (black cross). Then, the cell is lysed and a lysate containing SaPI-only particles is generated. These viral particles containing the synthetic SaPI with CRISPR-Cas9 cargo can be then deployed as antimicrobial elements to target and eliminate bacteria with the *mec*A sequence (yellow arrow). (b) All of the synthetic SaPIbov2 variants except the synthetic island with CRISPR-Cas9 targeting *rsa*E showed levels of transduction equal to the parent version induced under a background of packaging defective 80*α Δter*S. Graphs represent transduction titers performed in RN4220. Statistical analysis was performed using two-way ANOVA followed by Dunnett’s multiple comparisons test using the parent SaPIbov2 *bap*::*tet*M as control and as reference for all comparisons (n=4±SD). Adjusted p values for rebooted SaPIbov2 *bap*::*tet*M p=0.9976, SaPIbov2::*tet*M-*cas9*-∅ p=0.0846, SaPIbov2::*tet*M-*cas9-mec*A p=0.0745, SaPIbov2::*P_cad_-cas9-*∅*-tet*M p=0.0846, and SaPIbov2::*P_cad_-cas9-rsa*E-*tet*M ${}^{****}p<0.0001$. (c) A SaPIbov2 engineered with cadmium-inducible CRISPR-Cas9 system targeting *rsa*E was used to kill *S. aureus*. RN4220. RN4220 *Δrsa*E strain was used as control to show specific killing by target. Cadmium concentrations were used at 1 *μ*M. (d) Graphs represent transduction titers performed in RN4220. Statistical analysis was performed using two-way ANOVA followed by Dunnett’s multiple comparisons test using infection with SaPIbov2 *bap*::*tet*M as control for each strain and as reference for all comparisons (n=3±SD). Adjusted p values for infection of RN4220 *Δrsa*E with SaPIbov2::*P_cad_-cas9-*∅*-tet*M p=0.7317, and SaPIbov2::*P_cad_-cas9-rsa*E-*tet*M p=0.8916; and for infection of RN4220 with SaPIbov2::*P_cad_-cas9-*∅*-tet*M p=0.7248, and SaPIbov2::*P_cad_-cas9-rsa*E-*tet*M ${}^{****}p<0.0001$. (e) Killing of methicillin-resistant *S. aureus* USA100 and USA200 was performed by using synthetic SaPIbov2::*tet*M-*cas9* containing CRISPR-Cas9 targeting *mec*A. A SaPIbov2::*tet*M-*cas9* version with null (∅) gRNA was used as control. (f) Graphs represent transduction titers performed in MRSA strains. Statistical analysis was performed using two-way ANOVA followed by Dunnett’s multiple comparisons test using infection with SaPIbov2 *bap*::*tet*M as control for each strain and as reference for all comparisons (n=3±SD). Adjusted p values for infection of RN4220 with SaPIbov2::*tet*M-*cas9*-∅ p=0.9490, and SaPIbov2::*tet*M-*cas9-mec*A p=0.8988; for infection of USA100 with SaPIbov2::*tet*M-*cas9*-∅ p=0.8631, and SaPIbov2::*tet*M-*cas9-mec*A ${}^{****}p<0.0001$; for infection of USA200 with SaPIbov2::*tet*M-*cas9*-∅ p=0.9980, and SaPIbov2::*tet*M-*cas9-mec*A ${}^{****}p<0.0001$.

As previously mentioned, SaPIs have been recently proposed as an alternative to phages and antibiotics to combat *S. aureus* infections [15]. Due to their easy assembly in yeast and their high transferability *in vivo*, our method renders rapid engineering of synthetic SaPIs carrying different markers and antimicrobial payloads feasible. As a proof of concept, we therefore constructed several synthetic SaPIs carrying an antimicrobial payload (the CRISPR-Cas9 system [34, 35]) targeting either the methicillin resistance gene *mec*A or the conserved small regulatory RNA *rsa*E [36–38], to eliminate *S. aureus* cells (strains JP21059 and JP20282). As a control, we also generated a synthetic SaPI carrying the CRISPR-Cas9 system but no spacers against the *mec*A or *rsa*E genes (strains JP21058 and JP20281, respectively). SaPIbov2 was used here as scaffolding SaPI since it does not produce small SaPI capsids [39] and therefore, synthetic derivative SaPIs with an increased size (up to 45 kb, the size of the helper phage) can be efficiently packaged and transferred. Following the same workflow, we assembled SaPIbov2 with the two aforementioned cargos (see scheme in Figure S3). After their assembly in yeast, the synthetic SaPIs were rebooted into *S. aureus* lysogenic for the phage 80*α Δter*S mutant (JP12871). This recipient strain was used since after the induction of the mutant phage, the lysate will only contain engineered SaPIbov2 particles, but no phage particles [8, 29] (Figure 3(a)). We then proceeded to test the transduction rates of the engineered elements as well as the activity of the synthetic genes allocated in the adaptable module of SaPIbov2. When we used *S. aureus* RN4220 as a recipient strain, the transduction observed for the synthetic SaPIbov2 versions carrying either the CRISPR-Cas9 system with no spacers or with the gRNA targeting *mec*A was identical to that observed with a SaPIbov2 *bap*::*tet*M lysate (Figure 3(b)). Remarkably, very few transductants were obtained for the synthetic island carrying the inducible CRISPR-Cas9 system targeting *rsa*E, confirming the engineered island was able to kill *S. aureus* (Figures 3(c) and 3(d)). This was further confirmed using as recipient *S. aureus* RN4220 cells in which the targeted *rsa*E gene was removed (JP20283). Using these cells, the transfer of all the CRISPR-Cas9-containing island was identical to that observed for the SaPIbov2 *bap*::*tet*M (Figures 3(b) and S4).

The synthetic SaPI carrying the CRISPR-Cas9 targeting *mec*A was tested in two different scenarios: firstly, we tested its ability to cure a RN4220 derivative strain carrying a high-copy plasmid containing the cloned *mec*A resistance gene (strains JP17110) (Figure S5). Next, and having confirmed the activity of this island against this plasmid, we tested the ability of this synthetic island to kill MRSA strains. To this end, we used the MRSA USA100 and USA200 strains as recipients in this experiment (strains JP7581 and JP7593, respectively). As had previously occurred, the transduction of the synthetic SaPIbov2 targeting *mec*A was reduced 1000-fold in *S. aureus* USA100 and USA200 compared with RN4220 (Figures 3(e) and 3(f)), confirming that the CRISPR-Cas9 system present in the modified island was able to detect the target, cleave the double-stranded DNA, and trigger cell death.

**Figure 4:**
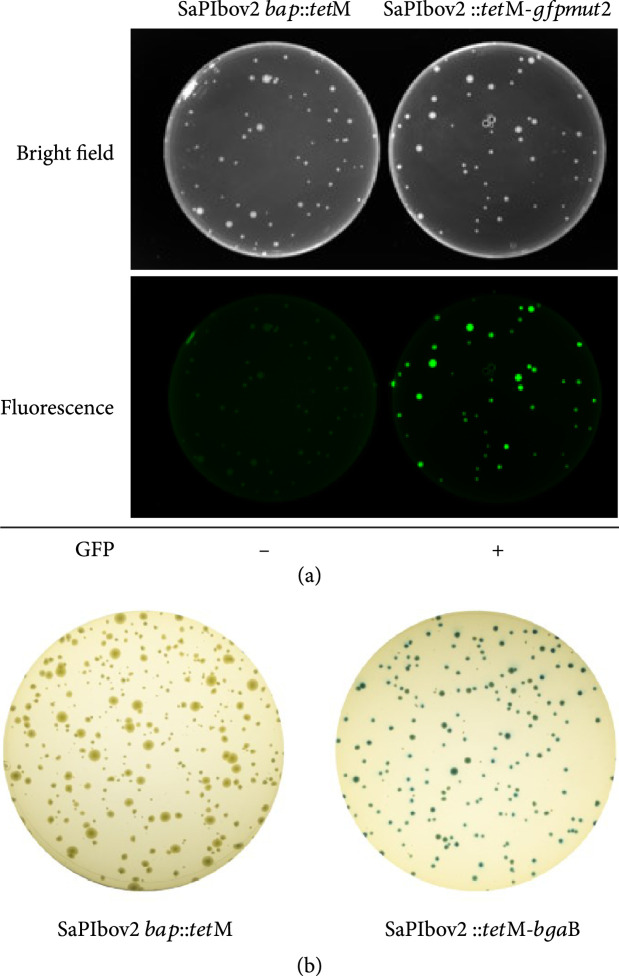
GFP and Beta-gal reporter SaPIs (a) GFP-mediated detection of *S. aureus* after infecting RN4220 cells with a lysates of 80*α Δter*S SaPIbov2 *tst*::*tet*M and 80*α Δter*S SaPIbov2::*tet*M-*gfpmut2* using MOI of 10. (b) Blue-white screening in X-gal plates was enabled by *β*-galactosidase expression in *S. aureus* RN4220 by transduction of the SaPIbov2::*tet*M-*bgaB* into recipient cells of RN4220. Cells transduced with SaPIbov2 *bap*::*tet*M were used as control.

Finally, we created SaPIbov2 derivatives carrying reporter genes (*gfpmut2* or *β*-galactosidase (*bga*B) (strains JP21056 and JP21057, respectively; see scheme in Figure S3) that would enable the use of PICIs as biosensors for bacterial diagnostics. The transfer of these islands by the 80*α* helper phage was undistinguishable to that observed for SaPIbov2 *bap*::*tet*M (Figure S6). Next, we tested the functionality of the reporter genes present in the synthetic islands. Plates exposed with Coomassie blue or fluorescein filter showed that all colonies carrying the SaPIbov2::*tet*M-*gfpmut2* island expressed GFP, whilst the control colonies carrying the wt SaPIbov2 *bap*::*tet*M did not show any fluorescence (Figure 4(a)). Similar results were obtained with the synthetic SaPIbov2 carrying the *bga*B gene. Thus, all the colonies were blue when grown in plates containing X-gal, while the control colonies carrying the wt SaPIbov2 *bap*::*tet*M island were white (Figure 4(b)). These SaPIbov2 variants used to express reporters can be further modified using specific promoters to act as biosensors for the detection of a specific genetic trait after infection. Taken together, these results validate our strategy for designing and rapidly rebooting SaPIs with diverse cargos that can be implemented for novel biotechnological purposes.

### 3.3. Assembly and Rebooting of Non-SaPI PICIs

Having assembled and rebooted different SaPIs, we sought to know whether this approach could be universalized as a strategy to assemble and reboot other PICIs. Following the strategy depicted in Figure 1, we tested whether it was possible to assemble in yeast the EcCICFT073 element from *E. coli* [3, 12]. Using three PCR fragments that covered the full EcCICFT073 element (including >500 bp of the chromosomal flanking sequence), we rebooted this PICI by transforming the YAC directly into *E. coli* strains C600 or 594. As *E. coli* is more readily transformable compared to *S. aureus*, the transformation of YAC-EcCICFT073 into *E. coli* gave rise to a significantly higher number of colonies per DNA unit (Figure S7). Next, we lysogenized the *E. coli* cells with the EcCICFT073 helper phages *λ* or *ϕ*80 [3, 12]. The resident prophages were induced with MC, and the cycle of the synthetic EcCICFT073 element analyzed. As shown in Figure S7, the transduction levels of the engineered element were indistinguishable from those observed for the wt PICI. In parallel to this, we rebooted another Gram-positive PICI, SaPIpT1028 into *S. aureus* (JP12871) to show that this technique can be employed to reboot other noncharacterized SaPI elements.

**Figure 5:**
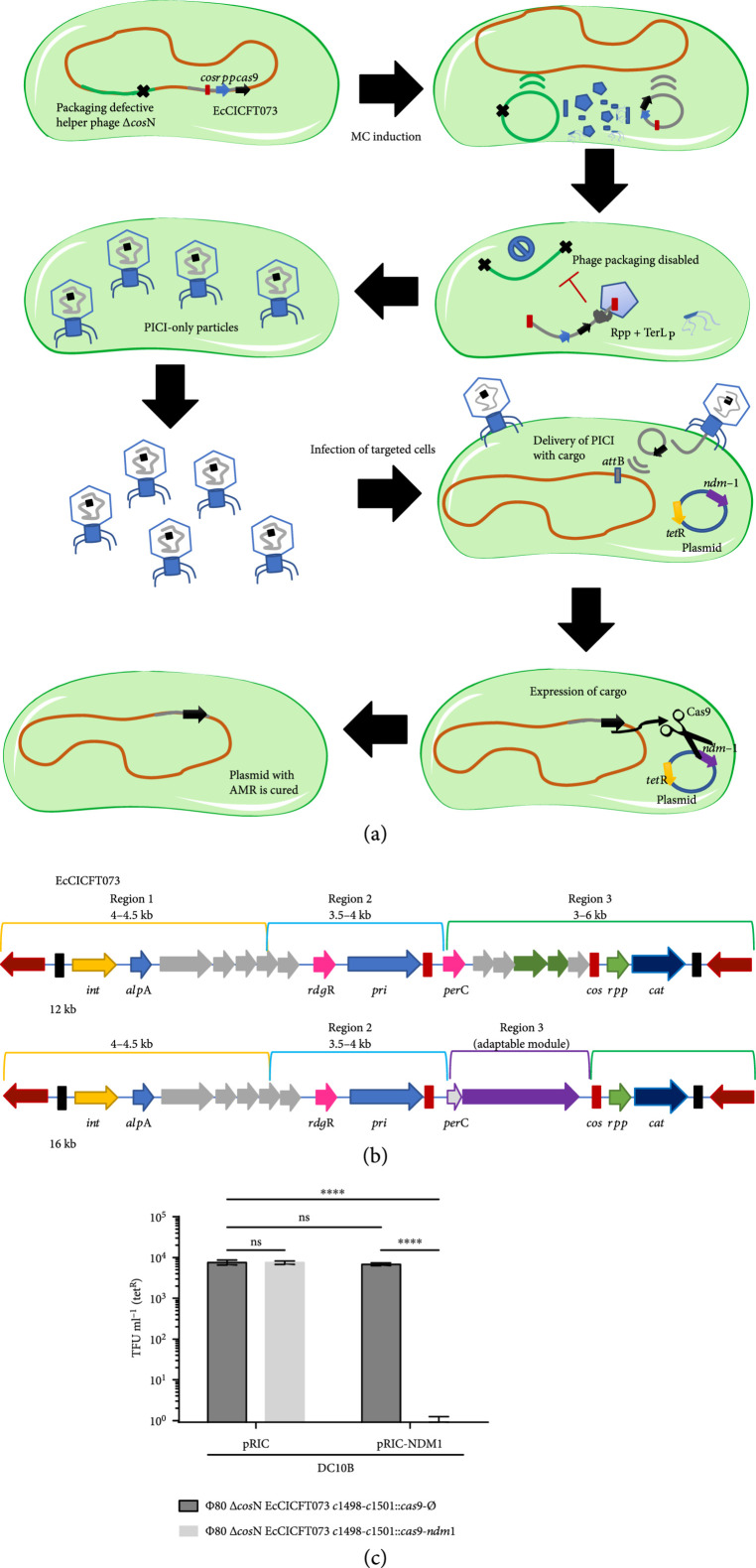
Synthetic Gram-negative PICI used as targeted therapy. (a) The synthetic Gram-negative PICI exploits the packaging machinery of its temperate phage to pack and deliver a CRISPR-Cas9 (black arrow) cargo into other bacteria. After induction with MC and activation of the ERP cycle, both phage (light green) and PICI (grey) excise from the bacterial (orange) chromosome to be packed into viral particles. Only the PICI-DNA is packed using the *cos* signaling (red block) to fulfil the viral particles hijacking the TerL of the phage with its cognate Rpp, while the phage-DNA packaging is disabled due to deletion of the *cos*N site (black cross). Then, the cell is lysed and a lysate containing PICI-only particles is generated. These viral particles containing the synthetic PICI with CRISPR-Cas9 cargo can be delivered as an antimicrobial element, to target and cure plasmids (blue) carrying virulence or AMR genes (purple and yellow arrows). (b) The synthetic EcCICFT073 *c1498-c1501*::*cas9* elements were designed by PCR fragments incorporating the CRISPR-Cas9 system in the adaptable module of the *cos* island. (c) Deployment and activity of Synthetic PICI with CRISPR-cas9 system against plasmid pRIC carrying the *ndm*-1 gene. Graphs represent the means of transduced cells with the chloramphenicol resistance cassette (*cat*) on tetracycline resistant cells (tet^R^) to measure the proportion of cells cured of pRIC1 with the target gene by transduction of the PICI. Statistical analysis was performed using one-way ANOVA followed by Tuckey’s multiple comparisons test (n=3±SD). Adjusted p values for pRIC *cas*9-∅ versus pRIC *cas*9-*ndm*1 p=0.729, pRIC *cas*9-∅ versus pRIC-*ndm*1 *cas*9-∅, p=0.6147, pRIC *cas*9-∅ versus pRIC-NDM1 *cas*9-*ndm*1 ${}^{****}p<0.0001$, and pRIC cas9-*ndm*1 versus pRIC-*ndm*1 *cas*9-*ndm*1 ${}^{****}p<0.0001$.

Finally, following our approach to develop synthetic SaPIs that can be used as Trojan horses to kill pathogenic bacteria, we also incorporated a CRISPR-Cas9 system that was previously employed to cure plasmids that confer antibiotic resistance in *E. coli* [27, 40] by targeting the New Delhi metallo-*β*-lactamase (NDM-1) gene. We tested the transduction and activity of the synthetic EcCICFT073 island producing Cas9 (strains JP20690 and JP20691) against cells carrying a plasmid with the Cas9-targeted sequence (JP17462) (Figure 5). To avoid the killing effect that could be produced by the helper phage, in this case, we transformed the YAC-EcCICFT073-Cas9 element into *E. coli* cells lysogenic for the phage *ϕ*80 *Δcos* mutant (strain JP17091). Since this prophage does not contain its cognate *cos* site, required for phage packaging, induction of this prophage produces a lysate that uniquely contains engineered EcCICFT073 elements (Figure 5(a)). As shown in Figure 5(c), the transfer of the synthetic EcCICFT073-Cas9 element removed the antibiotic resistance-conferring plasmid from the *E. coli* cells, confirming the efficiency of the synthetic EcCICFT073-Cas9 element and validating the strategy to use yeast to assemble and reboot synthetic PICIs.

## 4. Discussion

In this report, we have adapted a yeast-based platform for phage engineering to produce PICI elements *a la carte* with mutations and synthetic circuits. The current methods for genome engineering of these mobile genetic elements (MGEs) are laborious and can only produce a single mutation or knock-in at a time. Traditionally, the manipulation of SaPIs and other Gram-positive PICIs would be facilitated by allelic exchange [17, 24] or CRISPR-based methods [41–43] which have low frequencies of success and require several steps of screening in order to achieve the desired modification. In multiple cases, mutations may occur from sequences that are highly variable, and therefore, can generate frameshifts or stop codons of no interest. For manipulation of the Gram-negative PICIs, a *λ* red recombination system can be employed to create single mutations or deletions [16, 44]. However, this technique requires a strong selection marker and, in some cases, counterselection cannot be easily achieved because the element may already contain such a marker to track its transfer. Other methods, such as multiplex automated genome engineering (MAGE), could also be used for PICI engineering. However, MAGE relies heavily on the efficiency of the lambda recombinases, which works well in *E. coli* and a few Gram-negative bacteria [45, 46], but has not been validated in other bacterial. Surely, some recombinases could be employed for a similar application in *S. aureus* and other Gram-positive bacteria; however, our method can be applied as a generic platform that is independent of phage or host recombinases and only depends on the PICI being assembled and its own integration efficiency. An additional disadvantage of MAGE is that its efficiency diminishes substantially when attempting to introduce larger synthetic circuits (such as CRISPR-Cas9) from a single PCR product into the PICI. Moreover, assembling PICIs in yeast allows easy verification of the mutations prior to rebooting, and direct rebooting of PICIs in the host cells offers complete freedom for design and editing, including the incorporation of different modifications and elimination of any undesired features such as virulence factors, in a single assembly. Similar to the method employed by Ando et al. [20], we observed that assemblies in yeast had high rates of success. Whole-genome sequencing of strains JP20111, JP20117, JP20751, and JP21056 confirmed that none of the rebooted PICIs (SaPIbov1 *tst*::*tet*M, SaPIpT1028::*erm*C, SaPIbov2::*tet*M-*P_bla_-gfpmut2*, and SaPIbov2::*tet*M-*cas9-mec*A, respectively) were different to the template during the reassembly and rebooting process. Rebooting of the synthetic PICI DNA, mirroring its structure when integrated in the host cell, allowed us to avoid aberrant integrations in the yeast chromosomes and enabled the efficient excision and circularization of the element from the synthetic YAC and the posterior integration of the functional PICI in the bacterial chromosome. With high accuracy and reproducibility, this method can be employed as a tool to systematically investigate PICIs to comprehend their complex lifestyle.

We found that concentrations of the YAC-PICI DNA extracted from yeast had similar yields to the commonly used thermosensitive plasmids (pMAD, pBT2, pIMAY, etc.) for allelic replacement. Similarly, the successful transformation of these plasmids is strictly dependent on the quality of competent cells. In addition, rebooting Gram-negative PICIs was much easier than rebooting Gram-positive PICIs due to the efficiency of *E. coli* for acquiring plasmids (Figure S8). In addition to using diverse protocols for making electrocompetent cells, one can always opt to generate the YAC-PICI with the necessary elements for transformation in *E. coli* and then harvest a higher yield of DNA in order to attempt the transformation of the PICI in its host.

Our approach to assemble synthetic versions of the EcCICFT073 element not only demonstrates the engineering of the first synthetic Gram-negative PICI but also shows the incorporation of synthetic genes in a *cos* island, which needs to maintain a genome size equal to exact fractions of their helper phage genome (approx. 48 kb) (Figures 5(b) and 5(c)).

As shown by Ram et al. [15] and Kiga et al. [40], PICIs are a very effective delivery system for antimicrobial payloads because they possess higher frequency transfers than phagemids [27, 47, 48] and can be used to adapt larger modules than phages [49], having almost two-thirds of the size to spare for packaging DNA into the viral particles. Since PICIs depend on the induction of helper phages, they are unable to replicate and infect other bacteria after integration into the host genome and can no longer actively participate in the dissemination of other MGEs. Thus, in contrast to phages used to combat bacterial infections, PICIs on their own cannot contribute to the spread of virulence traits. As proof of principle, we employed two different constructs of the in SaPIbov2, one with an inducible promoter targeting *rsa*E and a constitutive promoter targeting *mec*A as options to be applied in different clinical scenarios. A constitutive promoter can be employed as a targeted therapy to directly treat wound infections, while the inducible promoter can be used as prophylaxis to efficiently eliminate bacteria complementing the administration of antibiotics (i.e., catheters). Overall, the applicability of PICIs as a tunable delivery system enables the use of different CRISPR-Cas systems with diverse promoters and the addition of other killing modules such as toxins, quorum sensing inhibitors, and lysins. Another advantage is that PICIs can use different helper phage chassis as a strategy to expand their host range, making them the perfect analogy of a Trojan horse to infiltrate and destroy pathogenic bacteria. In addition, one can employ different PICIs carrying diverse CRISPR arrays for a more efficient PICI cocktail. Thereby, we consider these elements as a safer platform for targeted antimicrobials compared to modified phage-based applications. Although there is a promising field to employ PICIs as a novel therapeutic approach, there are still aspects to be assessed concerning the use of genetically modified PICIs and their interaction with other pathogenicity islands found in clinical strains. In addition, to eliminate regulatory difficulties on their use as targeted therapy, PICIs with the CRISPR-Cas system will need to be designed without any antibiotic resistance cassette. In our experimental setting, the presence of the antibiotic markers facilitated the readout of the experiment, i.e., allowing us to identify the bacteria that were infected with the synthetic PICIs carrying or not the gRNA. However, this can be easily modified with our method.

Additionally, PICIs can be employed as an inexpensive and simpler tool for bacterial detection without the need of using DNA or RNA amplification, electrophoresis equipment, or expensive optical devices, as only bacterial culture plates are required. This approach benefits from the fact that the detection readout is produced by the pathogen itself, and its preparation requires little training. Reporter circuits in PICIs can be designed with specific promoters that will trigger the signal as a response to the expression of a specific gene related to biofilm formation, virulence, or other pathogenic traits. A simple portable device could be employed to detect bacteria infected by our synthetic PICIs, carrying either a CRISPR-Cas13a system [40] or different reporter genes, which might be observed directly after a period of incubation, allowing the sequential recovery of the pathogen for further investigation. Although this further highlights the benefits of using PICI-based detection, there are still some limitations such as (i) bacterial growth is required to use this approach; (ii) time will be required to amplify the readout signal; and (iii) specific promoters will be needed to differentiate between different bacterial strains.

In summary, our work shows that the rebooting of PICIs can be used as a rapid method to study these MGEs. We designed a strategy in which the PICIs are assembled in the YAC mirroring the structure they have when they are integrated into the bacterial chromosome to only promote their rebooting in their cognate host. We created synthetic PICIs with multiple mutations and adaptable modules in a faster and elegant manner than allelic replacement, allowing us to efficiently manipulate and reboot them in the host cells. Using this method, we will continue unveiling the complex biology and lifestyle of PICIs and their helper phages. We hope our method can be used by different MGE communities to systematically study them. Novel biotechnology approaches could arise following this top-down approach to deconstruct and manipulate viruses, to exploit them as a Trojan horse against pathogens, and to provide advantages over antibiotics.

## Additional Points

(i) An efficient and general method to generate and manipulate PICIs. (ii) Enable the study of PICIs by rapidly generating variants and mutations. (iii) Synthetic PICIs with adaptable modules for different biotechnology applications.

## Supplementary Materials

Supplementary Materials Table S1: strains used in this study. Table S2: plasmids used in this study. Table S3: oligonucleotides used in this study. Figure S1: schematics of PCR fragments for PICI assembly and process of excision and integration for rebooting. Mirroring the structure of the integrated elements, amplification of the first PCR fragment uses a forward primer that binds >500 bp outside the *att*L site, whilst the last PCR fragment uses a reverse primer that binds >500 bp outside the *att*R site. The overlap region to the YAC is highlighted in blue, and red represents the chromosome region before or after the *att* sites. Internal fragments are amplified with >30 bp overlaps to the adjacent PCR fragment (regions for regulation, packaging, and toxins are represented in yellow, green, and pink accordingly). Once the plasmid has been transformed into the host cell, this undergoes excision, leaving behind the YAC (blue) with the chromosomal region (red). The PICI then integrates in the corresponding *att*B site located in the genome. Figure S2: strategy used to generate SaPIbov1 *tst::tet*M mutants. Multiple PCR fragments were generated to introduce termination codons into *ppi*, *cpm*A, and *ter*S genes of SaPIbov1. Two amber stop codons were introduced in the primer design followed by a restriction site (XhoI, KpnI, and NotI accordingly) to identify the mutation of each gene. PCR fragments were combined to assemble the island with single, double, or triple mutations. Overlapping regions to the YAC (blue), chromosome (red), SaPI regulation module (yellow), packaging module (green), and toxin module (pink) are highlighted in each PCR product used to assemble the SaPI with mutations. Figure S3: design of synthetic cargos for SaPIbov2. PCR fragments were produced to maintain the essential genes for induction, replication, and packaging (region 1 to 3) of the SaPIbov2 element. A fragment containing the *tet*M cassette was used as region 4 to tract the transfer of the SaPI. Region 4 highlights the adaptable module were different synthetic constructs can be allocated in the island followed by the last fragment (region 5) containing the *att*R site to enable a successful integration of the synthetic islands. Figure S4: transduction titers of SaPIbov2 *::P*_*cad*_*-cas9-tet*M. Lysates of 80*α**Δter*S SaPIbov2*::P*_*cad*_*-cas9-tet*M with and without gRNA against *rsa*E were titered by infecting cultures of RN4220 and RN4220 *Δrsa*E. Cells with a concentration 1 *μ*M of CdCl_2_ were tested to further induce the expression of the CRISPR-Cas9 system in the island. Statistical analysis was performed using one-way ANOVA followed by Tukey’s multiple comparisons test (n=3±SD). Adjusted p values for wt *cas*9-∅ versus wt *cas*9-*rsa*E ${}^{***}p=0.0003$, *Δrsa*E *cas*9-∅ versus *Δrsa*E *cas*9-*rsa*E ns p>0.9999, wt *cas*9-∅ versus wt *cas*9-*rsa*E ${}^{****}p<0.0001$, and *Δrsa*E *cas*9-∅ versus *Δrsa*E *cas*9-*rsa*E ns p=0.8846. Figure S5: curing of high copy plasmid pCN51 carrying the *mec*A. Lysates of 80*α**Δter*S SaPIbov2*::tetM-cas*9 with and without a gRNA targeting the methicillin resistance gene *mec*A were assessed by infecting cultures of RN4220 carrying either the pCN51 empty plasmid or the pCN51-mecA at a cell density of ~10^5^ CFU ml^-1^ for (MOI of 10). Recovered cells were plated on TSA with erythromycin and tetracycline to measure the proportion of cells cured of pCN51 with the target. Statistical analysis was performed using one-way ANOVA followed by Tukey’s multiple comparisons test (n=3±SD). Adjusted p values for pCN51 *cas*9-∅ versus pCN51 *cas*9-*mec*A p=0.9975, pCN51 *cas*9-∅ versus pCN51-*mec*A *cas*9-∅, p=0.9868, pCN51 *cas*9-∅ versus pCN51-*mec*A *cas*9-*mec*A ${}^{****}p<0.0001$, and pCN51 *cas*9-*mec*A versus pCN51-*mec*A *cas*9-*mec*A ${}^{****}p<0.0001$. Figure S6: transfer of synthetic SaPIbov2 with reporters. The synthetic SaPIbov2 variants with reporters SaPIbov2*::tet*M-*gfpmut2* and SaPIbov2*::tetM-bgaB* showed levels of transduction equal to the parent version induced under a background of packaging defective 80*α*. Statistical analysis was performed using two-way ANOVA followed by Dunnett’s multiple comparisons test (n=3±SD ns p>0.9999) with the parent strain 80*α**Δter*S SaPIbov2 *bap::tet*M as control. Figure S7: assembly and rebooting of non-SaPI PICIs. Assembly and rebooting of non-SaPI PICIs. Transduction titers of rebooted PICIs EcCICFT073 and SaPIpT1028 were compared to their wt counterparts. The *E. coli* EcCICFT073 element was induced and transferred using phages *λ* and *ϕ*80. The *S. aureus* element SaPIpT1028 induced and packed by phage 80*α**Δter*S. Graphs represent transduction titers performed on recipient strains. Statistical analysis was performed using one-way ANOVA followed by Tukey’s multiple comparisons test (n=4±SD). Adjusted p values for transduction titers of *λ* EcCICFT073 wt (JP13413) versus rebooted (JP20123) p=0.9994, *ϕ*80 EcCICFT073 wt (JP13410) versus rebooted (JP20124) p=0.8307, 80*α**Δter*S SaPIpT1028 wt (JP21184) versus rebooted (JP210117) p>0.9999. Figure S8: transformation efficiency of YAC-PICIs used in this study. Electrocompetent cells of *S. aureus* or *E. coli* were electroporated with 2 *μ*g of YAC-PICI DNA assembled and extracted from yeast. Graphs showed at least three independent replicates from each transformation [3, 6, 12, 17, 23–27, 30, 40, 41, 48, 51–57].
