# Supplementary material for: Rebooting Synthetic Phage-Inducible Chromosomal Islands: One Method to Forge Them All
Source: Biodes Res. 2020 May 11;2020:5783064. doi: 10.34133/2020/5783064 (PMC10530653; doi:10.34133/2020/5783064)
Supplement: Supplementary Materials — Table S1: strains used in this study. Table S2: plasmids used in this study. Table S3: oligonucleotides used in this study. Figure S1: schematics of PCR fragments for PICI assembly and process of excision and integration for rebooting. Mirroring the structure of the integrated elements, amplification of the first PCR fragment uses a forward primer that binds >500 bp outside the attL site, whilst the last PCR fragment uses a reverse primer that binds >500 bp outside the attR site. The overlap region to the YAC is highlighted in blue, and red represents the chromosome region before or after the att sites. Internal fragments are amplified with >30 bp overlaps to the adjacent PCR fragment (regions for regulation, packaging, and toxins are represented in yellow, green, and pink accordingly). Once the plasmid has been transformed into the host cell, this undergoes excision, leaving behind the YAC (blue) with the chromosomal region (red). The PICI then integrates in the corresponding attB site located in the genome. Figure S2: strategy used to generate SaPIbov1 tst::tetM mutants. Multiple PCR fragments were generated to introduce termination codons into ppi, cpmA, and terS genes of SaPIbov1. Two amber stop codons were introduced in the primer design followed by a restriction site (XhoI, KpnI, and NotI accordingly) to identify the mutation of each gene. PCR fragments were combined to assemble the island with single, double, or triple mutations. Overlapping regions to the YAC (blue), chromosome (red), SaPI regulation module (yellow), packaging module (green), and toxin module (pink) are highlighted in each PCR product used to assemble the SaPI with mutations. Figure S3: design of synthetic cargos for SaPIbov2. PCR fragments were produced to maintain the essential genes for induction, replication, and packaging (region 1 to 3) of the SaPIbov2 element. A fragment containing the tetM cassette was used as region 4 to tract the transfer of the SaPI. Region 4 highlights the adapta [file 5783064.f1.zip › Supplementary Materials.docx]

**Supplementary Materials**

**Tables S1-S3**

| **Table S1. Strains used in this study** | | |  |
| --- | --- | --- | --- |
| **Specie** | **Strain** | **Description** | **Reference** |
| *E. coli* | **DC10B** | *mcrA* Δ(*mrr*-*hsdRMS*-*mcrBC*) φ80*lacZ*ΔM15 Δ*lacX*74 *recA*1 *araD*139 Δ(*ara-leu*)7697 *galU* *galK* *rpsL* *endA1* *nupG* Δ*dcm* | (*38*) |
| *S. aureus* | **RN450** | NCTC8325 cured of φ11, φ12 and φ13 | (*51*) |
| *S. aureus* | **RN4220** | RN450 restriction-defective | (*52*) |
| *S. aureus* | **RN10616** | RN4220 80α | (*6*) |
| *S. aureus* | **RN451** | RN450 φ11 | (*51*) |
| *S. aureus* | **JP7581** | HA-VR-MRSA USA100/CC5 SCCmec II | (*53*) |
| *S. aureus* | **JP7593** | HA-VR-MRSA USA200/CC30 SCCmec II | (*54*) |
| *S. aureus* | **JP6399** | RN4420 80α erythromycin marked | (*55*) |
| *S. aureus* | **JP6400** | RN4220 φ11 erythromycin marked | (*56*) |
| *S. aureus* | **JP1996** | RN4220 SaPIbov1 *tst*::*tet*M | (*57*) |
| *S. aureus* | **JP2129** | RN4420 SaPIbov2 *bap*::*tet*M | (*58*) |
| *S. aureus* | **JP3603** | RN10359 80α SaPIbov1 *tst*::*tet*M | (*25*) |
| *S. aureus* | **JP12871** | RN4220 80α ⍙*ter*S | (*35*) |
| *S. aureus* | **JP13894** | RN4220 pCN51 | This study |
| *S. aureus* | **JP17110** | RN4220 pCN51-*mec*A | This study |
| *S. aureus* | **JP20283** | RN4220 ∆*rsa*E | This study |
| *S. aureus* | **JP20284** | JP20283 80α ∆*ter*S::*erm*C | This study |
| *S. aureus* | **JP21184** | JP12871 SaPIpT1028 ::*erm*C | This study |
| *E. coli* | **594** | Laboratory strain derivative from K-12 ATCC10798 |  |
| *E. coli* | **C600** | ATCC23724, NCIB10222, F- *sup*E44 *lac*Y1 *thr*-1 *leu*B6 *mcr*A thi-1 *rfb*D1 *fhu*A21 | |
| *E. coli* | **CFT073** | clinical isolate ATCC700928, ø1, ø2, ø4, ø5 |  |
| *E. coli* | **JP10400** | C600 𝝺 |  |
| *E. coli* | **JP12507** | 594 φ80 |  |
| *E. coli* | **JP13410** | JP12507 EcCICFT073 *c1504-c1507*::*cat* | (*3*) |
| *E. coli* | **JP13413** | JP10400 EcCICFT073 *c1504-c1507*::*cat* | (*12*) |
| *E. coli* | **JP17091** | JP12507 ∆*cos*N | This study |
| *E. coli* | **JP20124** | JP17091 pBAD-*alp*A | This study |
| *E. coli* | **JP17459** | DC10B pRC319-*ndm*1 | This study |
| *E. coli* | **JP17584** | DC10B pRC319-null | This study |
| *E. coli* | **JP17120** | DC10B pRIC | This study |
| *E. coli* | **JP17121** | DC10B pRIC1 | This study |
| *E. coli* | **JP17122** | DC10B pRIC2 | This study |
| *E. coli* | **JP17123** | DC10B pRIC3 | This study |
| *E. coli* | **JP17124** | DC10B pRIC4 | This study |
| *E. coli* | **JP17125** | DC10B pRIC5 | This study |
| *E. coli* | **JP17451** | DC10B pRIC10 | This study |
| *E. coli* | **JP17454** | DC10B pRIC13 | This study |
| *E. coli* | **JP17462** | DC10B pRIC-*ndm*1 | This work |
| *S. cerevisiae* | **BY23849** | MATa *leu*2Δ0 *ura*3Δ0 *his*3-Δ1 *met*15Δ0 | Toh-e, Akio:Research Center for Pathogenic Fungi Chiba University |
| *S. cerevisiae* | **JP20109** | BY23849 YAC-SaPIbov1 *tst*::*tet*M | This study |
| *S. aureus* | **JP20111** | RN10616 YAC-SaPIbov1 *tst*::*tet*M | This study |
| *S. cerevisiae* | **JP20115** | BY23849 YAC-pT1028 ::*erm*C | This study |
| *S. aureus* | **JP20117** | JP12871 YAC-pT1028 ::*erm*C | This study |
| *S. cerevisiae* | **JP20122** | BY23849 YAC-EcCICFT073 *c1504-c1507*::*cat* | This study |
| *E. coli* | **JP20123** | JP10400 YAC-EcCICFT073 *c1504-c1507*::*cat* | This study |
| *E. coli* | **JP20124** | JP12507 YAC-EcCICFT073 *c1504-c1507*::*cat* | This study |
| *S. cerevisiae* | **JP20533** | BY23849 YAC-EcCICFT073 *c1498-c1501*::*Ptet-cas*9-null *c1504-c1507*::*cat* | This study |
| *S. cerevisiae* | **JP20534** | BY23849 YAC-EcCICFT073 *c1498-c1501*::*Ptet-cas*9-ndm1 *c1504-c1507*::*cat* | This study |
| *E. coli* | **JP20690** | JP20124 YAC-EcCICFT073 *c1498-c1501*::*Ptet-cas*9-null *c1504-c1507*::*cat* | This study |
| *E. coli* | **JP20691** | JP20124 YAC-EcCICFT073 c1498-c1501::*Ptet-cas*9-ndm1 *c1504-c1507*::*cat* | This study |
| *S. cerevisiae* | **JP20279** | BY23849 YAC-SaPIbov2 ::*Pcad-cas9*-null-*tet*M | This study |
| *S. cerevisiae* | **JP20280** | BY23849 YAC-SaPIbov2 ::*Pcad-cas9*-*rsa*E-*tet*M | This study |
| *S. aureus* | **JP20281** | JP20284 YAC-SaPIbov2 ::*Pcad-cas*9-null-*tet*M | This study |
| *S. aureus* | **JP20282** | JP20284 YAC-SaPIbov2 ::*Pcad-cas*9-*rsa*E-*tet*M | This study |
| *S. cerevisiae* | **JP20733** | BY23849 YAC-SaPIbov1 *ter*S* *tst*::*tet*M | This study |
| *S. cerevisiae* | **JP20735** | BY23849 YAC-SaPIbov1 *ppi***cpm*A* *tst*::*tet*M | This study |
| *S. cerevisiae* | **JP20737** | BY23849 YAC-SaPIbov1 *ppi***cpm*A* *ter*S* *tst*::*tet*M | This study |
| *S. cerevisiae* | **JP20741** | BY23849 YAC-SaPIbov2 *bap*::*tet*M | This study |
| *S. cerevisiae* | **JP20743** | BY23849 YAC-SaPIbov2 ::*tet*M-*Pbla-gfpmut*2 | This study |
| *S. cerevisiae* | **JP20745** | BY23849 YAC-SaPIbov2 ::*tet*M-*Pbla-bga*B | This study |
| *S. cerevisiae* | **JP20749** | BY23849 YAC-SaPIbov2 ::*tet*M-*cas*9-null | This study |
| *S. cerevisiae* | **JP20751** | BY23849 YAC-SaPIbov2 :*tet*M-*cas*9-mecA | This study |
| *S. aureus* | **JP20777** | RN4220 YAC-SaPIbov1 *tst*::*tet*M | This study |
| *S. aureus* | **JP20779** | RN4220 YAC-SaPIbov1 *ter*S* *tst*::*tet*M | This study |
| *S. aureus* | **JP20781** | RN4220 YAC-SaPIbov1 *ppi***cpm*A* *tst*::*tet*M | This study |
| *S. aureus* | **JP20783** | RN4220 YAC-SaPIbov1 *ppi***cpm*A* *ter*S* *tst*::*tet*M | This study |
| *S. aureus* | **JP21055** | JP12871 YAC-SaPIbov2 *bap*::*tet*M | This study |
| *S. aureus* | **JP21056** | JP12871 YAC-SaPIbov2 ::*tet*M-*Pbla-gfpmut*2 | This study |
| *S. aureus* | **JP21057** | JP12871 YAC-SaPIbov2 ::*tet*M-*Pbla-bga*B | This study |
| *S. aureus* | **JP21058** | JP12871 YAC-SaPIbov2 ::*tet*M-*cas*9-null | This study |
| *S. aureus* | **JP21059** | JP12871 YAC-SaPIbov2 ::*tet*M-*cas*9-mecA | This study |
| *S. aureus* | **JP21212** | JP6399 SaPIbov1 *tst*::*tet*M | This study |
| *S. aureus* | **JP21213** | JP6399 SaPIbov1 *ter*S* *tst*::*tet*M | This study |
| *S. aureus* | **JP21214** | JP6399 SaPIbov1 *ppi***cpm*A* *tst*::*tet*M | This study |
| *S. aureus* | **JP21215** | JP6399 SaPIbov1 *ppi***cpm*A* *ter*S* *tst*::*tet*M | This study |
| *S. aureus* | **JP21216** | JP6400 SaPIbov1 *tst*::*tet*M | This study |
| *S. aureus* | **JP21217** | JP6400 SaPIbov1 *ter*S* *tst*::*tet*M | This study |
| *S. aureus* | **JP21218** | JP6400 SaPIbov1 *ppi***cpm*A* *tst*::*tet*M | This study |
| *S. aureus* | **JP21219** | JP6400 SaPIbov1 *ppi***cpm*A* *ter*S* *tst*::*tet*M | This study |

| **Table S2. Plasmids used in this study.** | |  |
| --- | --- | --- |
| **Plasmid** | **Description** | **Reference** |
| **pBR322** | AmpR, TetR, ColE1 ori, bla. Cloning vector | (*48*) |
| **pBAD-alpA** | AmpR. alpA Expression vector | (*3*) |
| **pMAD** | AmpR, ColE1 ori, bgaB, ermC, temperature-sensitive replicon for Gram-positive bacteria (repBCAD)e | (*17*) |
| **pCN68** | AmpR, pT181 replicon, ColE1 ori, ermC, PblaZ-gfpmut2 Expression vector. Shuttle vector | (*50*) |
| **pCN51** | AmpR, pT181 replicon, ColE1 ori, ermC, Pcad-cadC Expression vector. Shuttle vector | (*50*) |
| **pIMAY** | CmR, ColE1 ori, Phelp-cat, anti-secY, temperature-sensitive replicon for Gram-positive bacteria (repBCAD)e | (*38*) |
| **pDB114** | CmR, CRISPR-Cas9 expression vector in *S. aureus*, rep from pC194 | (*45*) |
| **pFREE** | TetR, ColE1 ori Ptet-CRISPR-Cas9 expression vector | (*49*) |
| **pRC319** | KanR, Ptet-CRISPR-Cas9 expression vector, pBR322 ori, F1 ori, crRNA for blaNDM-1 | (*36*) |
| **pKLC26** | CmR, Target gene expression vector, p15A ori, intl1 promoter, ndm-1 | (*36*) |
| **pCN51-mecA** | pCN51 mecA GenBank: QHN12945.1 | (*37*) |
| **pRIC** | pBR322 ori, tetR pBR322, araC | This study |
| **pRIC-ndm1** | pRIC ndm-1 GenBank: FN396876 | This study |
| **pRIC1** | pRIC ColE1 ori, Phelp-cat pIMAY | This study |
| **pRIC2** | pRIC1 BsaI sites from pDB114 | This study |
| **pRIC3** | pRIC2 pT181 replicon from pCN51 | This study |
| **pRIC4** | pRIC3 cas9 and trcr mRNA from pDB114 | This study |
| **pRIC5** | pRIC4 ∆pT181 replicon | This study |
| **pRIC10** | pRIC5 crRNA from pRC319 | This study |
| **pRIC13** | pRIC10 mecA spacers | This study |
| **pAUR112** | Yeast artificial chromosome (YAC) AmpR, ColE1 ori, CEN/ARS, URA3, AUR1-C, Shuttle vector | Takara Bio Cat No 3601 |
| **pRIC76** | CEN/ARS, URA3 YAC-SaPIbov1 tst::tetM | This study |
| **pRIC78** | CEN/ARS, URA3 YAC-pT1028 ::ermC | This study |
| **pRIC81** | CEN/ARS, URA3YAC-EcCICFT073 c1503-c1507::cat | This study |
| **pRIC86** | CEN/ARS, URA3 YAC-EcCICFT073 c1498-c1501::Ptet-cas9-null c1504-c1507::cat | This study |
| **pRIC87** | CEN/ARS, URA3 YAC-EcCICFT073 c1498-c1501::Ptet-cas9-ndm1 c1504-c1507::cat | This study |
| **pRIC88** | CEN/ARS, URA3 YAC-SaPIbov2 ::Pcad-cas9-null-tetM | This study |
| **pRIC89** | CEN/ARS, URA3 YAC-SaPIbov2 ::Pcad-cas9-rsaE-tetM | This work |
| **pRIC103** | CEN/ARS, URA3 YAC-SaPIbov1 tst::tetM | This study |
| **pRIC104** | CEN/ARS, URA3 YAC-SaPIbov1 terS* (Q8*) tst::tetM | This study |
| **pRIC105** | CEN/ARS, URA3 YAC-SaPIbov1 ppi* (K2*) cpmA* (K7*) tst::tetM | This study |
| **pRIC106** | CEN/ARS, URA3 YAC-SaPIbov1 ppi* (K2*) cpmA* (K7*) terS* (Q8*) tst::tetM | This study |
| **pRIC108** | CEN/ARS, URA3 YAC-SaPIbov2 bap::tetM | This study |
| **pRIC109** | CEN/ARS, URA3 YAC-SaPIbov2 ::tetM-Pbla-gfpmut2 | This study |
| **pRIC110** | CEN/ARS, URA3 YAC-SaPIbov2 ::tetM-Pbla-bgaB | This study |
| **pRIC112** | CEN/ARS, URA3 YAC-SaPIbov2 ::tetM-cas9-null | This study |
| **pRIC113** | CEN/ARS, URA3 YAC-SaPIbov2 ::tetM-cas9-mecA | This study |

| **Table S3. Oligonucleotides used in this study** | | |  |
| --- | --- | --- | --- |
| **Plasmid** | **Primers** | **Sequence (5’-3’)** | **Source** |
| **pBAD-alpA** |  |  | **(*3*)** |
| **pMAD** |  |  | **(*17*)** |
| **pCN68** |  |  | **(*50*)** |
| **pCN51** |  |  | **(*50*)** |
| **pCN51-mecA** | **pCN51-mecA-1F** | AGAGGATCCAAAATTTCATCTTACAACTAATGAAACAGA | **This study** |
|  | **pCN51-mecA-2R** | CGCCTGAATTCCGATTTTATAACTTGTTTTATCGTCTAA |  |
| **pRC319** |  |  | **(*36*)** |
| **pKLC26** |  |  | **(*37*)** |
| **pBR322** |  |  | **(*48*)** |
| **pRIC** | **pBR322-ori-1F** | GTCATAAAGATCTTGCTTTCCATAGGCTCCGCCCC | **This study** |
|  | **pBR322-tet-6R** | GTAACTGCAGGTTCTCCGCAAGAATTGATTGGC |  |
|  | **pBAD-araC-PstI-1F** | ATGGCTGCAGTTATGACAACTTGACGGC |  |
|  | **pBAD-araC-BglII-2R** | GCAAGATCTATGGCTGAAGCGCA |  |
| **pRIC-ndm1** |  |  | **This study** |
|  |  |  |  |
| **pIMAY** |  |  | **(*38*)** |
| **pRIC1** | **AatII-pBR322ori-1F** | ACGACGTCGTTTTTCCATAGGCTCCG | **This study** |
|  | **KasI-pBR322ori-2R** | ATAAGGCGCCAGTTTTCGTTCCACTGAG |  |
|  | **KasI-CmR-1F** | AACTGGCGCCGTTAGTGACATTAGAAAACCGACTG |  |
|  | **AatII-CmR-2R** | CCATGACGTCGTAATACGACTCACTATAGGGC |  |
| **pDB114** |  |  | **(*45*)** |
| **pFREE** |  |  | **(*49*)** |
| **pRIC2** | **XbaI-BsaIsites-1F** | ACTAGTTCTAGAGTTTTAGAGCTATGCTGTTTTG | **This study** |
|  | **NotI-BsaIsites-2R** | GTGGCGGCCGCGTTTTGGGACCATTCAAAAC |  |
| **pRIC3** | **ApaI-repStaph-1F** | ACCGGGCCCCGGGAGGTCAGCTGTTAG | **This study** |
|  | **XhoI-repStaph-2R** | CGACCTCGAGGGAAGGATCCAAATCACAG |  |
| **pRIC4** | **XhoI-cas9-1F** | ACGACTCGAGGGATTAAGAGATTAATTTCCCTAAAAATG | **This study** |
|  | **SalI-cas9-2R** | CTTGTCGACGAAATAATCTTCATCTAAAATATACTTCAGTCACC |  |
| **pRIC5** | **ApaI-repStaph-1F** | ACCGGGCCCCGGGAGGTCAGCTGTTAG | **This study** |
|  | **XhoI-repStaph-2R** | CGACCTCGAGGGAAGGATCCAAATCACAG |  |
| **pRIC10** | **SalI-crRNA-1F** | TTCGTCGACTATTTCTTAATAACTAAAAATATGG | **This study** |
|  | **XbaI-crRNA-2R** | AACTCTAGACTCGTAGACTATTTTTGTC |  |
| **pRIC13** | **mecA-crRNA1-F** | AAACCTATAAAGATGATGCAGTTATTGGTAAAAAG | **This study** |
|  | **mecA-crRNA1-R** | AAAACTTTTTACCAATAACTGCATCATCTTTATAG |  |
|  | **mecA-crRNA2-F** | AAACTGGCTCAGGTACTGCTATCCACCCTCAAACG |  |
|  | **mecA-crRNA2-R** | AAAACGTTTGAGGGTGGATAGCAGTACCTGAGCCA |  |
| **pAUR112** |  |  | **Takara Bio Cat No 3601** |
| **pRIC79** | **SaPIbov1-YAC-frag1-F** | TTCGAGAAAGTGCCCATCAGTGGGAGAACGACAAATATGGAAATGGC | **This study** |
| **YAC-SaPIbov1 tst::tetM** | **SaPIbov1-frag1-R** | GCGTTCAGTGATTTGAGGGATTGTAC |  |
|  | **SaPIbov2-frag2-F** | CTCTTCTAAAGATGGTGCATTTCTCAC |  |
|  | **SaPIbov2-frag2-R** | CCTAATTTATCGTGTACAGTTTGTCC |  |
|  | **SaPIbov1-frag3-F** | GGTGAATGGGACGGAGACGATG |  |
|  | **SaPIbov1-YAC-frag4-R** | GCGTTGCTGGCGTTTTTCCATAGGTTACAAATTCCCGGTAACCATTCC |  |
|  | **YAC-SaPIbov1-3F** | TCAAGAGAATGAAGCCGGAATGGTTACCGGGAATTTGTAACCTATGGAAAAACGCCAGCAACG |  |
|  | **YAC-SaPIbov1-2R** | ACTCTTGTTCTTTTGCCATTTCCATATTTGTCGTTCTCCCACTGATGGGCACTTTCTCGAA |  |
| **pRIC104** | **SaPIbov1-YAC-frag1-F** | TTCGAGAAAGTGCCCATCAGTGGGAGAACGACAAATATGGAAATGGC | **This study** |
| **YAC-SaPIbov1 terS* tst::tetM** | **SaPIbov1-frag1-R** | GCGTTCAGTGATTTGAGGGATTGTAC |  |
|  | **SaPIbov1-frag2-F** | GGTTTTTATCACAAGTACAATCCCTCAAATCAC |  |
|  | **SaPIbov1-terSstop-frag-R** | GCACTTTGTGTAACATTAAGTGTTCTAATATACTCATTCGCGGCCGCCTACTATTTTGCCGTTAACTCACTCATTCTATC |  |
|  | **SaPIbov1-terSstop-frag-F** | GATAGAATGAGTGAGTTAACGGCAAAATAGTAGGCGGCCGCGAATGAGTATATTAGAACACTTAATGTTACACAAAGTGC |  |
|  | **SaPIbov1-YAC-frag4-R** | GCGTTGCTGGCGTTTTTCCATAGGTTACAAATTCCCGGTAACCATTCC |  |
|  | **YAC-SaPIbov1-3F** | TCAAGAGAATGAAGCCGGAATGGTTACCGGGAATTTGTAACCTATGGAAAAACGCCAGCAACG |  |
|  | **YAC-SaPIbov1-2R** | ACTCTTGTTCTTTTGCCATTTCCATATTTGTCGTTCTCCCACTGATGGGCACTTTCTCGAA |  |
| **pRIC105** | **SaPIbov1-YAC-frag1-F** | TTCGAGAAAGTGCCCATCAGTGGGAGAACGACAAATATGGAAATGGC | **This study** |
| **YAC-SaPIbov1 ppi*cpmA* tst::tetM** | **SaPIbov1-frag1-R** | GCGTTCAGTGATTTGAGGGATTGTAC |  |
|  | **SaPIbov1-frag2-F** | GGTTTTTATCACAAGTACAATCCCTCAAATCAC |  |
|  | **SaPIbov1-ppistop-frag-R** | CTTTTACATAATCCTCGAGCTACTATTTAAGTTGTTCTTTATCCATGTGTAACCTCCAGCATTAATTTGG |  |
|  | **SaPIbov1-ppistop-frag-F** | GGTTACACATGGATAAAGAACAACTTAAATAGTAGCTCGAGGATTATGTAAAAGAATATAAGGAGATACCGATATATC |  |
|  | **SaPIbov1-cpmAs-frag2-R** | TCTTTAAACTCGGTACCCTACTACATTTTTATCCCCTTTACACTTCAATTCGTTTCAAAG |  |
|  | **SaPIbov1-cpmAs-frag2-F** | GGATAAAAATGTAGTAGGGTACCGAGTTTAAAGAATATAATCAATTTGTATTAGATCAACAC |  |
|  | **SaPIbov1-frag2-R** | CTGCCTTATCTAACTCCTCATCGTCTCC |  |
|  | **SaPIbov1 frag3-F** | GGTGAATGGGACGGAGACGATG |  |
|  | **SaPIbov1-YAC-frag4-R** | GCGTTGCTGGCGTTTTTCCATAGGTTACAAATTCCCGGTAACCATTCC |  |
|  | **YAC-SaPIbov1-3F** | TCAAGAGAATGAAGCCGGAATGGTTACCGGGAATTTGTAACCTATGGAAAAACGCCAGCAACG |  |
|  | **YAC-SaPIbov1-2R** | ACTCTTGTTCTTTTGCCATTTCCATATTTGTCGTTCTCCCACTGATGGGCACTTTCTCGAA |  |
| **pRIC106** | **SaPIbov1-YAC-frag1-F** | TTCGAGAAAGTGCCCATCAGTGGGAGAACGACAAATATGGAAATGGC | **This study** |
| **YAC-SaPIbov1 ppi*cpmA* terS* tst::tetM** | **SaPIbov1-frag1-R** | GCGTTCAGTGATTTGAGGGATTGTAC |  |
|  | **SaPIbov1-frag2-F** | GGTTTTTATCACAAGTACAATCCCTCAAATCAC |  |
|  | **SaPIbov1-ppistop-frag-R** | CTTTTACATAATCCTCGAGCTACTATTTAAGTTGTTCTTTATCCATGTGTAACCTCCAGCATTAATTTGG |  |
|  | **SaPIbov1-ppistop-frag-F** | GGTTACACATGGATAAAGAACAACTTAAATAGTAGCTCGAGGATTATGTAAAAGAATATAAGGAGATACCGATATATC |  |
|  | **SaPIbov1-cpmAs-frag2-R** | TCTTTAAACTCGGTACCCTACTACATTTTTATCCCCTTTACACTTCAATTCGTTTCAAAG |  |
|  | **SaPIbov1-cpmAs-frag2-F** | GGATAAAAATGTAGTAGGGTACCGAGTTTAAAGAATATAATCAATTTGTATTAGATCAACAC |  |
|  | **SaPIbov1-terSstop-frag-R** | GCACTTTGTGTAACATTAAGTGTTCTAATATACTCATTCGCGGCCGCCTACTATTTTGCCGTTAACTCACTCATTCTATC |  |
|  | **SaPIbov1-terSstop-frag-F** | GATAGAATGAGTGAGTTAACGGCAAAATAGTAGGCGGCCGCGAATGAGTATATTAGAACACTTAATGTTACACAAAGTGC |  |
|  | **SaPIbov1-YAC-frag4-R** | GCGTTGCTGGCGTTTTTCCATAGGTTACAAATTCCCGGTAACCATTCC |  |
|  | **YAC-SaPIbov1-3F** | TCAAGAGAATGAAGCCGGAATGGTTACCGGGAATTTGTAACCTATGGAAAAACGCCAGCAACG |  |
|  | **YAC-SaPIbov1-2R** | ACTCTTGTTCTTTTGCCATTTCCATATTTGTCGTTCTCCCACTGATGGGCACTTTCTCGAA |  |
| **pRIC108** | **SaPIbov1-YAC-frag1-F** | TTCGAGAAAGTGCCCATCAGTGGGAGAACGACAAATATGGAAATGGC | **This study** |
| **YAC-SaPIbov2 bap::tetM** | **SaPIbov2-frag1-R** | GAGGATTTTATTTTCGAGGGATCTATTG |  |
|  | **SaPIbov2-frag2-F** | CTCTTCTAAAGATGGTGCATTTCTCAC |  |
|  | **SaPIbov2-frag2-R** | CCTAATTTATCGTGTACAGTTTGTCC |  |
|  | **SaPIbov2-frag3-F** | GCTGGTGATGGTTATAGTGAAGTT |  |
|  | **SaPIbov2-frag3-R** | CGAGATTTGGGTTGCCTTTGTTTCTTGATCTGTAGCATGTATTGTGATAGC |  |
|  | **TetM-SaPIbov2-F** | GCTATCACAATACATGCTACAGATCAAGAAACAAAGGCAACCCAAATC |  |
|  | **SaPIbov1-YAC-frag4-R** | GCGTTGCTGGCGTTTTTCCATAGGTTACAAATTCCCGGTAACCATTCC |  |
|  | **YAC-SaPIbov1-3F** | TCAAGAGAATGAAGCCGGAATGGTTACCGGGAATTTGTAACCTATGGAAAAACGCCAGCAACG |  |
|  | **YAC-SaPIbov1-2R** | ACTCTTGTTCTTTTGCCATTTCCATATTTGTCGTTCTCCCACTGATGGGCACTTTCTCGAA |  |
| **pRIC88 and pRIC89** | **Sb2-Fragment1_Rv** | TTCGAGAAAGTGCCCATCAGTGGGAGAACGACAAATATGGAAATGGC | **This study** |
| **YAC-SaPIbov2 ::Pcad-cas9-tetM** | **Sb2-Fragment2_Fw** | GAGGATTTTATTTTCGAGGGATCTATTG |  |
|  | **Sb2-Fragment2_Rv** | CTCTTCTAAAGATGGTGCATTTCTCAC |  |
|  | **Sb2-Fragment3_Fw** | CCTAATTTATCGTGTACAGTTTGTCC |  |
|  | **Sb2-Fragmento3_Rv2 (Pcd)** | AGTGCGCATGGTCGACCTGGTGTAGCATGTATTGTGATAGC |  |
|  | **PCad_SalI_Fw** | CCAGGTCGACCATGCGCACTTATTCAAGTG |  |
|  | **Pcad_Rv** | AGGTTCAGACATTGACCTTCAC |  |
|  | **cas9_Fw** | GTGAAGGTCAATGTCTGAACCTACTAGTTTTAGGAGGATGATTATTTATGGATAAGAAATACTCAATAGGC |  |
|  | **cas9_Rv** | AAACGGGTCTTGAGGGGTTTTTTGAGATCTGGTACCCGGGTTAATTAAGTTGCGCACACCGACTAGCG |  |
|  | **SmaI-PacI-tracrRNA_pFREE_Rv** | CCCGGGTTAATTAAgttgcgcacaccgactagcg |  |
|  | **SP01_gRNA(rsaE)_tracrRNA_Fw** | CTGCAGTTGACAAATTGCAGTAGGCATGACAAAATGGACTCAGGGAGAAATTTTTCACTTCAAACAAAGGTTTTAGAGCTAGAAATAGCAAGTTAAAATAAGGCTAGTC |  |
|  | **Sb2-Fragmento tetM_Fw** | CCTACTGCAATTTGTCAACTGCAGGTATCGATAAGCTTGATATCG |  |
|  | **Sb2-ragmento Tet_Rv** | GCTCTAGAACTAGTGGATCCCC |  |
|  | **Sb2-Fragment5_Fw2** | GGGGATCCACTAGTTCTAGAGCGGGGATATTATGGTATGAATTTTTC |  |
| **pRIC112 and pRIC113** | **SaPIbov1-YAC-frag1-F** | TTCGAGAAAGTGCCCATCAGTGGGAGAACGACAAATATGGAAATGGC | **This study** |
| **YAC SaPIbov2 ::tetM-cas9** | **SaPIbov2-frag1-R** | GAGGATTTTATTTTCGAGGGATCTATTG |  |
|  | **SaPIbov2-frag2-F** | CTCTTCTAAAGATGGTGCATTTCTCAC |  |
|  | **SaPIbov2-frag2-R** | CCTAATTTATCGTGTACAGTTTGTCC |  |
|  | **SaPIbov2-frag3-F** | GCTGGTGATGGTTATAGTGAAGTT |  |
|  | **SaPIbov2-frag3-R** | CGAGATTTGGGTTGCCTTTGTTTCTTGATCTGTAGCATGTATTGTGATAGC |  |
|  | **TetM-SaPIbov2-F** | GCTATCACAATACATGCTACAGATCAAGAAACAAAGGCAACCCAAATC |  |
|  | **SaPIbov1-frag3-2R** | CGTCTTGGCGTCTGGC |  |
|  | **SaPIbov1-cas9-frag3-F** | AATAACAATCAAAGAGCCAGACGCCAAGACGGGATTAAGAGATTAATTTCCCTAAAAATG |  |
|  | **SaPIbov1-cas9-frag3-R** | CGTATTCACTGCCTGCAACGTGTGGGATGATACGGTAATACGACTCACTATAGGGC |  |
|  | **SaPIbov1-frag4-F** | GTATCATCCCACACGTTGCAG |  |
|  | **SaPIbov1-YAC-frag4-R** | GCGTTGCTGGCGTTTTTCCATAGGTTACAAATTCCCGGTAACCATTCC |  |
|  | **YAC-SaPIbov1-3F** | TCAAGAGAATGAAGCCGGAATGGTTACCGGGAATTTGTAACCTATGGAAAAACGCCAGCAACG |  |
|  | **YAC-SaPIbov1-2R** | ACTCTTGTTCTTTTGCCATTTCCATATTTGTCGTTCTCCCACTGATGGGCACTTTCTCGAA |  |
| **pRIC109** | **SaPIbov1-YAC-frag1-F** | TTCGAGAAAGTGCCCATCAGTGGGAGAACGACAAATATGGAAATGGC | **This study** |
| **YAC SaPIbov2 ::tetM-Pbla-gfpmut2** | **SaPIbov2-frag1-R** | GAGGATTTTATTTTCGAGGGATCTATTG |  |
|  | **SaPIbov2-frag2-F** | CTCTTCTAAAGATGGTGCATTTCTCAC |  |
|  | **SaPIbov2-frag2-R** | CCTAATTTATCGTGTACAGTTTGTCC |  |
|  | **SaPIbov2-frag3-F** | GCTGGTGATGGTTATAGTGAAGTT |  |
|  | **SaPIbov2-frag3-R** | CGAGATTTGGGTTGCCTTTGTTTCTTGATCTGTAGCATGTATTGTGATAGC |  |
|  | **TetM-SaPIbov2-F** | GCTATCACAATACATGCTACAGATCAAGAAACAAAGGCAACCCAAATC |  |
|  | **SaPIbov1-frag3-2R** | CGTCTTGGCGTCTGGC |  |
|  | **gfpmut2-SaPIbov1-frag3-F** | CAAAGAGCCAGACGCCAAGACGGCATGCAGCTTACTATGCCATTATT |  |
|  | **gfpmut2-SaPIbov1-frag3-R** | CTGCAACGTGTGGGATGATACTTATTTGTATAGTTCATCCATGCCA |  |
|  | **SaPIbov1-frag4-F** | GTATCATCCCACACGTTGCAG |  |
|  | **SaPIbov1-YAC-frag4-R** | GCGTTGCTGGCGTTTTTCCATAGGTTACAAATTCCCGGTAACCATTCC |  |
|  | **YAC-SaPIbov1-3F** | TCAAGAGAATGAAGCCGGAATGGTTACCGGGAATTTGTAACCTATGGAAAAACGCCAGCAACG |  |
|  | **YAC-SaPIbov1-2R** | ACTCTTGTTCTTTTGCCATTTCCATATTTGTCGTTCTCCCACTGATGGGCACTTTCTCGAA |  |
| **pRIC110** | **SaPIbov1-YAC-frag1-F** | TTCGAGAAAGTGCCCATCAGTGGGAGAACGACAAATATGGAAATGGC | **This study** |
| **YAC SaPIbov2 ::tetM-Pbla-bgaB** | **SaPIbov2-frag1-R** | GAGGATTTTATTTTCGAGGGATCTATTG |  |
|  | **SaPIbov2-frag2-F** | CTCTTCTAAAGATGGTGCATTTCTCAC |  |
|  | **SaPIbov2-frag2-R** | CCTAATTTATCGTGTACAGTTTGTCC |  |
|  | **SaPIbov2-frag3-F** | GCTGGTGATGGTTATAGTGAAGTT |  |
|  | **SaPIbov2-frag3-R** | CGAGATTTGGGTTGCCTTTGTTTCTTGATCTGTAGCATGTATTGTGATAGC |  |
|  | **TetM-SaPIbov2-F** | GCTATCACAATACATGCTACAGATCAAGAAACAAAGGCAACCCAAATC |  |
|  | **SaPIbov1-frag3-2R** | CGTCTTGGCGTCTGGC |  |
|  | **bgaB-SaPIbov1-frag3-3F** | CAAAGAGCCAGACGCCAAGACGTGTCTAGTTAATGTGTAACGTAACATTAGC |  |
|  | **bgaB-SaPIbov1-frag3-2R** | CTGCAACGTGTGGGATGATACCTAAACCTTCCCGGCTTCATC |  |
|  | **SaPIbov1-frag4-F** | GTATCATCCCACACGTTGCAG |  |
|  | **SaPIbov1-YAC-frag4-R** | GCGTTGCTGGCGTTTTTCCATAGGTTACAAATTCCCGGTAACCATTCC |  |
|  | **YAC-SaPIbov1-3F** | TCAAGAGAATGAAGCCGGAATGGTTACCGGGAATTTGTAACCTATGGAAAAACGCCAGCAACG |  |
|  | **YAC-SaPIbov1-2R** | ACTCTTGTTCTTTTGCCATTTCCATATTTGTCGTTCTCCCACTGATGGGCACTTTCTCGAA |  |
| **pRIC78** | **pT1028-YAC-frag1-F** | TTCGAGAAAGTGCCCATCAGTGGAACGTGGATTTATCCGTGCCG | **This study** |
| **YAC-pT1028 ::ermC** | **pT1028-frag1-R** | CGTGAGTCTAAACTTTCATAATGTACCACTG |  |
|  | **pT1028-frag2-F** | GGCGAGGAATCAGATGACGC |  |
|  | **pT1028-frag2-R** | ACAAAGTTCGCACACCTTGC |  |
|  | **pT1028-frag3-F** | GGTGTTAGCTTTTAAAATCGGAAGG |  |
|  | **pT1028-YAC-frag3-2R** | GCGTTGCTGGCGTTTTTCCATAGGGGGTTTGCATTGAATTCATGCAACTAGG |  |
|  | **YAC-3F** | CCTATGGAAAAACGCCAGCAACG |  |
|  | **YAC-2R** | CACTGATGGGCACTTTCTCGAA |  |
| **pRIC781** | **CFT073-YAC-frag1-F** | GAATTTCGAGAAAGTGCCCATCAGTGCCACTCGCCAGCGACGAC | **This study** |
| **YAC-EcCICFT073 c1504-c1507::cat** | **CFT073-frag1-R** | CGAACTGTGTCCGGTCTTTCTGTTCTTC |  |
|  | **CFT073-frag2-F** | CTTGCGCAGGGAGAAGAACAGAAAGAC |  |
|  | **CFT073-frag2-R** | AACCTGATGCCGTTCCTTGTCTGTCTC |  |
|  | **CFT073-frag3-F** | CATGCAACTGGTGGAGACAGACAAGG |  |
|  | **CFT073-YAC-frag3-R** | GTTGCTGGCGTTTTTCCATAGGCCCGGCAACATCGCGG |  |
|  | **YAC-CFT073-3F** | TTATCATTCAGGGTACCGGTCGTCGTCGCTGGCGAGTGGCACTGATGGGCACTTTCTCGAAAT |  |
|  | **YAC-CFT073-2R** | AGAACGCAGCGTACTTTGCAGCACCGCCAGTGCCTATGGAAAAACGCCAGCAACG |  |
| **pRIC86 and pRIC87** | **CFT073-YAC-frag1-F** | GAATTTCGAGAAAGTGCCCATCAGTGCCACTCGCCAGCGACGAC | **This study** |
| **YAC-EcCICFT073 c1498-c1501::Ptet-cas9 c1504-c1507::cat** | **CFT073-frag1-R** | CGAACTGTGTCCGGTCTTTCTGTTCTTC |  |
|  | **CFT073-frag2-F** | CTTGCGCAGGGAGAAGAACAGAAAGAC |  |
|  | **CFT073-cas9-frag2-2R** | TTCAAAACGCGTCGACTTGCTGGGTCCCGCTCAGAACGGAATATCATCACCGTAAGG |  |
|  | **cas9-CFTfrag3-1F** | CGGGAGGCGATGACCCTTACGGTGATGATATTCCGTTCTGAGCGGGACCAGCAAGTCGACGCGTTTTG |  |
|  | **cas9-CFTfrag3-2R** | AACGGGTCCATATGCCGGAAAGGACCCATAAAAAAAGCCGGATTTCTCCGGCCTTAATCATCATCTAGAGAATGGAGACC |  |
|  | **CFT073-cas9-frag3-2F** | AGAGCTATGCTGTTTTGAATGGTCTCCATTCTCTAGATGATGATTAAGGCCGGAGAAATCCGGC |  |
|  | **CFT073-YAC-frag3-R** | GTTGCTGGCGTTTTTCCATAGGCCCGGCAACATCGCGG |  |
|  | **YAC-CFT073-3F** | TTATCATTCAGGGTACCGGTCGTCGTCGCTGGCGAGTGGCACTGATGGGCACTTTCTCGAAAT |  |
|  | **YAC-CFT073-2R** | AGAACGCAGCGTACTTTGCAGCACCGCCAGTGCCTATGGAAAAACGCCAGCAACG |  |
| **Colony PCR** | **Primers** | **Sequence (5’-3’)** | **Source** |
| **Check SaPIbov1 ORF5 (terS)** | **SaPIbov1-ORF5-F** | GGTAGAGGTGATAGAATGAGTGAG | **This study** |
|  | **Tet-3cE** | CCGGAATTCTGTCTATGATGTTCACCTTCG |  |
| **Check SaPIbov1 ORF9 (cpmA)** | **SaPIbov1-ORF8-R** | GCCTACCCGAATTGGATAG |  |
|  | **SaPIbov1-ORF10-F** | GGGGTGTAGAAATGGAAAGTATC |  |
| **Check SaPIbov1 ORF12 (ppi)** | **SaPIbov1-ORF12-R** | CCTGCTCGTGTTTCAAATTG |  |
|  | **SaPIbov1-ORF14-F** | GGGGCTTTGTATGGAAACAG |  |
| **Check SaPIbov1 ORF21 (int)** | **SaPIbov1-112mE** | CCGGAATTCAATTGCTGAGGCAAAACTTC |  |
|  | **SaPIbov-att-9cE** | CCGGAATTCATTGAGTGGGAATAATTATATATAGC |  |
| **Check SaPIbov1 (attR)** | **SaPIbov1-frag4-F** | GAAAAATATGTATCATCCCACACG |  |
|  | **SaPIbovI- 116 mP** | AAAACTGCAGCAATGAAATAAGTGTTTTTCGTTAG |  |
| **Check SaPIbov2** | **SaPIbov2-frag2-F** | CTCTTCTAAAGATGGTGCATTTCTCAC |  |
|  | **SaPIbov2-frag1-R** | GAGGATTTTATTTTCGAGGGATCTATTG |  |
| **Check cas9 in SaPIbov2** | **TetM-check-F** | AGCAGAGCTAAAAGGATATCAGG |  |
|  | **Cas9-check-R** | TCCACTGTCAAATAAAAGAGCC |  |
|  | **Cas9-check-F** | GCTGGCTAGTGCCGGAG |  |
|  | **SaPIbov1-check-YAC-R** | CGTATTCACTGCCTGCAACGTG |  |
| **Check gfpmut2 in SaPIbov2** | **TetM-check-F** | AGCAGAGCTAAAAGGATATCAGG |  |
|  | **GFP-SpeI-2R** | ATCACTAGTTTATTTGTATAGTTCATCCATGCCA |  |
| **Check bgaB in SaPIbov2** | **TetM-check-F** | AGCAGAGCTAAAAGGATATCAGG |  |
|  | **bgal-2c** | TGGCCATTGCTCTGGGTTATAAT |  |
| **Check mecA spacers in SaPIbov2** | **Cas9-check-F** | GCTGGCTAGTGCCGGAG |  |
|  | **mecA-crRNA2-R** | AAAACGTTTGAGGGTGGATAGCAGTACCTGAGCCA |  |
| **Check SaPIpT1028** | **pT1028-31mB** | CGCGGATCCCTGAAGTTGTGTGTGATAGCG |  |
|  | **pT1028-4cBg** | GAAGATCTCATGCTATAAATCACTTCATTC |  |
|  | **SaPIn130-mS** | ACGCGTCGACATTGATAATGAAATGGTGCGC |  |
|  | **SaPIn1-102m** | AAAATAACCTTTCACATGCCC |  |
|  | **orf007-pT1028-2c** | AAAATATACATCCCACGTATC |  |
|  | **SaPIbov1-ORF10-1mB** | CGCGGATCCTACACAAGAGGATTACAAGGC |  |
| **Check EcCICFT073** | **EcoCFT073-int-7mE** | CCGGAATTCTGTGATTTTCCTTGTTCCAGC |  |
|  | **EcoCFT073-int-9cB** | CGCGGATCCACCAATGTGCTGTATTACGTG |  |
|  | **EcoCFT-c1488-1mS** | ACGCGTCGACACAGGAAGCACGCCAGCAATC |  |
|  | **EcoCFT-c1494-2cS** | ACGCGTCGACACTACCCGCAAAAATAATGAC |  |
| **Check cas9 in EcCICFT073** | **Cas9-check-F** | GCTGGCTAGTGCCGGAG |  |
|  | **EcoCFT-c1503-4R** | CCAGACTTTCCGCGATCCG |  |
|  | **EcoCFT-c1497-1F** | AGATTGCCGAAGCGGAACAGG |  |
|  | **Cas9-check-R** | TCCACTGTCAAATAAAAGAGCC |  |
| **Check ndm1 gene** | **NDM-1-F** | ACAAAATCCATTCTCATGGAATTGCCCAATATTATG |  |
|  | **NDM-1-R** | CGAATTCTCAGCGCAGCTTGTCGGC |  |
| **Southern blot** | **Primers** | **Sequence (5’-3’)** | **Source** |
| **tetM probe** | **tetM_F** | GTGGACAAAGGTACAACGAGG | **This study** |
|  | **tetM_R** | CTTTCCTCTTGTTCGAGTTCC |  |

**Supplementary Figures S1-S7**

**Figure S1. Schematics of PCR fragments for PICI assembly and process of excision and integration for rebooting.** Mirroring the structure of the integrated elements, amplification of the first PCR fragment uses a forward primer that binds >500 bp outside the *att*L site, whilst the last PCR fragment uses a reverse primer that binds >500 bp outside the *att*R site. The overlap region to the YAC is highlighted in blue, red represents the chromosome region before or after the *att* sites. Internal fragments are amplified with >30 bp overlaps to the adjacent PCR fragment (regions for regulation, packaging and toxins are represented in yellow, green and pink accordingly). Once the plasmid has been transformed into the host cell, this undergoes excision, leaving behind the YAC (blue) with the chromosomal region (red). The PICI then integrates in the corresponding *att*B site located in the genome.

**Figure S2. Strategy used to generate SaPIbov1 *tst*::*tet*M mutants.** Multiple PCR fragments were generated to introduce termination codons into *ppi, cpm*A and *ter*S genes of SaPIbov1. Two amber stop codons were introduced in the primer design followed by a restriction site (XhoI, KpnI and NotI accordingly) to identify the mutation of each gene. PCR fragments were combined to assemble the island with single, double or triple mutations. Overlapping regions to the YAC (blue), chromosome (red), SaPI regulation module (yellow), packaging module (green), and toxin module (pink) are highlighted in each PCR product used to assemble the SaPI with mutations.

**Figure S3. Design of synthetic cargos for SaPIbov2.** PCR fragments were produced to maintain the essential genes for induction, replication and packaging (region 1 to 3) of the SaPIbov2 element. A fragment containing the *tet*M cassette was used as region 4 to tract the transfer of the SaPI. Region 4 highlights the adaptable module were different synthetic constructs can be allocated in the island followed by the last fragment (region 5) containing the *att*R site to enable a successful integration of the synthetic islands.

**Figure S4. Transduction titres of SaPIbov2 *::P_cad_-cas9*-*tet*M.** Lysates of 80α Δ*ter*S SaPIbov2 *::P_cad_-cas9*-*tet*M with and without gRNA against *rsa*E were tittered by infecting cultures of RN4220 and RN4220 Δ*rsa*E. Cells with a concentration 1 μM of CdCl_2_ were tested to further induced the expression of the CRISPR-Cas9 system in the island. Statistical analysis was performed using one-way ANOVA followed by Tuckey’s multiple comparisons test (n=3 ±SD). Adjusted p-values for wt *cas*9-∅ versus wt *cas*9-*rsa*E ***p=0.0003, Δ*rsa*E cas9-∅ versus Δ*rsa*E *cas*9-*rsa*E ns p> 0.9999, wt *cas*9-∅ versus wt *cas*9-*rsa*E ****p< 0.0001, and Δ*rsa*E cas9-∅ versus Δ*rsa*E *cas*9-*rsa*E ns p=0.8846.

**Figure S5. Curing of high copy plasmid pCN51 carrying the *mec*A.** Lysates of 80α Δ*ter*S SaPIbov2 ::*tet*M-*cas9* with and without a gRNA targeting the methicillin resistance gene *mec*A were assessed by infecting cultures of RN4220 carrying either the pCN51 empty plasmid or the pCN51-mecA at a cell density of ~10^5^ CFU ml^-1^ for (MOI of 10). Recovered cells were plated on TSA with erythromycin and tetracycline to measure the proportion of cells cured of pCN51 with the target. Statistical analysis was performed using one-way ANOVA followed by Tuckey’s multiple comparisons test (n=3 ±SD). Adjusted p-values for pCN51 *cas*9-∅ versus pCN51 *cas*9-*mec*A p=0.9975, pCN51 *cas*9-∅ versus pCN51-*mec*A *cas*9-∅, p=0.9868, pCN51 cas9-∅ versus pCN51-*mec*A *cas*9-*mec*A ****p< 0.0001, and pCN51 *cas*9-*mec*A versus pCN51-*mec*A cas9-*mec*A ****p< 0.0001.

**Figure S6. Transfer of synthetic SaPIbov2 with reporters.** The synthetic SaPIbov2 variants with reporters SaPIbov2 ::*tet*M-*gfpmut2* and SaPIbov2 ::*tet*M-*bgaB* showed levels of transduction equal to the parent version induced under a background of packaging defective 80α. Statistical analysis was performed using two-way ANOVA followed by Dunnett’s multiple comparisons test (n=3 ±SD ns p> 0.9999) with the parent strain 80α Δ*ter*S SaPIbov2 *bap*::*tet*M as control.

**Figure S7. Assembly and rebooting of non-SaPI PICIs.** Assembly and rebooting of non-SaPI PICIs. Transduction titers of rebooted PICIs EcCICFT073 and SaPIpT1028 were compared to their wt counterparts. The *E. coli* EcCICFT073 element was induced and transfer using phages λ and φ80. The *S. aureus* element SaPIpT1028 induced and packed by phage 80α Δ*ter*S*.* Graphs represent transduction titers performed on recipient strains. Statistical analysis was performed using one-way ANOVA followed by Tuckey’s multiple comparisons test (n=4 ±SD). Adjusted p-values for transduction titers of λ EcCICFT073 wt (JP13413) versus rebooted (JP20123) p=0.9994, φ80 EcCICFT073 wt (JP13410) versus rebooted (JP20124) p=0.8307, 80α Δ*ter*S SaPIpT1028 wt (JP21184) vs rebooted (JP210117) p> 0.9999.

**Figure S8. Transformation efficiency of YAC-PICIs used in this study*.*** Electro-competent cells of *S. aureus* or *E. coli* were electroporated with 2 μg of YAC-PICI DNA assembled and extracted from yeast. Graphs showed at least three independent replicates from each transformation.

**Supplementary Materials and Methods**

**Yeast assembly method (to be submitted in bio-protocol.org)**

1. Inoculate *S. cerevisiae* strain BJ5464 into 20 ml of appropriate media and incubate at 30 °C, 210 RPM overnight. Yeast without a plasmid (such as BJ5464) can be grown in 2X YPAD.
2. On a 500 ml flask with 100 ml of 2X YPAD inoculate 4 ml of overnight culture. This should make up to 20 transformations.
3. Incubate at 30 °C, 210 RPM until the cell titer has reached 2.0 x 10^7^ cells ml^-1^ or OD600 1.0. At this point, cells will have performed two divisions.
4. For each transformation, harvest 3-4 ml of cells by centrifugation at max speed (> 6,000 x g) for 30 sec.
5. Once 3-4 ml were harvest, resuspend cells in 1.0 M LiAc, centrifuge at max speed for 30 sec and discard supernatant.
6. Wash cells twice following step number 5. Make sure to discard all the supernatant using pipette.
7. Cells can also be frozen and stored using storing solution (5% glycerol, 10% DMSO) and incubating them in Mr. Freeze for 4 h at -80°C.
8. Add the following ingredients directly to the cell pellet in the order they are listed. Carrier DNA needs to be at room temperature for 5 minutes prior to use.
   Add ~250 ng of YAC PCR and ~500 ng of each PCR fragment.

*Note – the volume can exceed the 14 µl listed below without any problems.

| **Ingredients** | **1 X** |
| --- | --- |
| PEG 50% w/v | 260 µl |
| LiAc 1.0 M | 36 µl |
| Single-stranded carrier DNA (2 mg ml^-1^) | 50 µl |
| DNA + ddH_2_O | 14 µl |
| **Total volume** | **360** µl |

1. Vortex vigorously to re-suspend cells for 2 min.
2. Incubate at 42 °C for 45 min. Mix periodically by inversion.
3. Centrifuge at > 6,000 x g for 3 min and remove supernatant.
4. Resuspend pellet in 200 µl ddH_2_O. Careful when resuspending – use pipette tip to mix cells. DO NOT VORTEX.
5. Plate all the volume onto appropriate synthetic dropout media (SD) plates. The goal is to get several hundred of transformants.
6. Incubate at 30 °C for 3-5 days.
7. Pick 15 colonies and re-streak them onto SD media plates and incubate at 30 °C for next day.
8. Perform Yeast colony PCR using primers from one region to the other in order to generate a product that proves the gap-repair between two different PCR fragments.
9. From each isolated colony, smear with a 10 µl tip on an Eppendorf tube and microwave the tube with the smeared yeast for 5 min. Place the Eppendorf tubes at the edges of the microwave plate.
10. Incubate tubes on ice for 5 min and then add PCR reaction to each sample.
11. Once positive colonies have been identified, inoculate 20 ml of SD media on a 100 ml flask and incubate for 48 h at 30 °C and 210 rpm.
12. Spin down cultures at > 6,000 x g for 10 min.
13. Resuspend cultures into 500 µl of lyticase buffer (1 M sorbitol and 0.1M Na2EDTA pH 7.5) and split into two tubes, then add 20 µl of lyticase solution (Lyticase 2.5 µg µl^-1^, 1.2 M sorbitol and 10mM sodium phosphate pH 7.5) to each tube.
14. Incubate samples for 2 h at 37 °C and 120 rpm.
15. Spin down cultures at > 6,000 x g for 3 min and discard supernatant with a tip.
16. Proceed with Qiagen miniprep extraction kit protocol and elute plasmids in <30 µl of MiliQ H_2_O.
17. Measure plasmid concentration and determine how many ug have been obtained. Generally, >1 µg of plasmid DNA is used for a successful transformation in both *E.coli*  and *S. aureus* electro-competent cells.
18. For PICI rebooting, transformed competent cells and after electroporation incubate 2 h at 37 °C and 120 rpm. Then, spin down cells (5 min at 8000 rpm) and plate the pellet in solid media with antibiotic.
19. For phage rebooting, transformed competent cells and after electroporation dilute 1/50 into TSB + Phage buffer (1 mM NaCl, 0.05 M Tris pH 7.8, 1 mM MgSO4, 4 mM CaCl2) to increase the quantity of rebooted phage and enable its detection. For phages with antibiotic markers, centrifuged the overnight culture and plate on media with antibiotic.

**SD Media** 1 liter 500 ml 200 ml

Yeast nitrogen base 6.7 g 3.35 g 1.34 g
(with ammonium sulfate)

20X Dropout Solution 50 ml 25 ml 10 ml

100X Supplements 10 ml 5 ml 2 ml

Agar 20 g 10 g 4 g

Add After Autoclaving

40% Glucose 50 ml 25 ml 10 ml

| **100X amino acid solution**  L-Histidine HCl: 200 mg / 100ml L-Leucine: 1 g / 100 ml  L-Tryptophan: 200 mg / 100 ml | **20X Dropout Solution (per liter)**  L-Isoleucine 300 mg L-Valine 1500 mg L-Phenylalanine 500 mg L-Arginine HCL 200 mg L-Tyrosine 300 mg L-Threonine 300 mg L-Lysine 300 mg L-Methionine 200 mg L-Adenine hemisulfate 600 mg |
| --- | --- |

**1.0 M Lithium acetate**

1 M Lithium acetate, pH 7.5

**50% PEG:**

50 g per 100 ml (MW= 3,350)

**2X YPAD Media 250 ml 500 ml 1 L**

2 % yeast extract 5 g 10 g 20 g

4% peptone 10 g 20 g 40 g

4% glucose 10 g 20 g 40 g

Adenine hemisulfate 20 mg 40 mg 80 mg

**YPD stock media comes with 10g of yeast extract, 20g of peptone and 20g of glucose per litre. 50 g are used for 500 ml of 2X YPD media

**Lyticase Buffer 200 ml**

0.1M Na2EDTA pH 7.5 7.44 g

1M Sorbitol 36.434 g

**Lyticase Solution 4 ml**

Lyticase or Zymolase 2.5 mg/ml

1.2 M sorbitol 3.96 ml

1 M sodium phosphate pH 7.5 40 ul

*first make buffer, then add 4 ml to vial
